# The Disequilibrium of Nucleosomes Distribution along Chromosomes Plays a Functional and Evolutionarily Role in Regulating Gene Expression

**DOI:** 10.1371/journal.pone.0023219

**Published:** 2011-08-19

**Authors:** Peng Cui, Qiang Lin, Lingfang Zhang, Feng Ding, Chengqi Xin, Daoyong Zhang, Fanglin Sun, Songnian Hu, Jun Yu

**Affiliations:** 1 The CAS Key Laboratory of Genome Sciences and Information, Beijing Institute of Genomics, Chinese Academy of Sciences, Beijing, China; 2 Institute of Epigenetics and Cancer Research, School of Medicine, Tsinghua University, Beijing, China; 3 Computational Bioscience Research Centre, King Abdullah University of Science and Technology, Thuwal, Kingdom of Saudi Arabia; 4 Graduate School of the Chinese Academy of Sciences, Beijing, China; King Abdullah University of Science and Technology, Saudi Arabia

## Abstract

To further understand the relationship between nucleosome-space occupancy (NO) and global transcriptional activity in mammals, we acquired a set of genome-wide nucleosome distribution and transcriptome data from the mouse cerebrum and testis based on ChIP (H3)-seq and RNA-seq, respectively. We identified a nearly consistent NO patterns among three mouse tissues—cerebrum, testis, and ESCs—and found, through clustering analysis for transcriptional activation, that the NO variations among chromosomes are closely associated with distinct expression levels between house-keeping (HK) genes and tissue-specific (TS) genes. Both TS and HK genes form clusters albeit the obvious majority. This feature implies that NO patterns, i.e. nucleosome binding and clustering, are coupled with gene clustering that may be functionally and evolutionarily conserved in regulating gene expression among different cell types.

## Introduction

The nucleosome, as the basic unit of eukaryotic chromatin, consists of a histone core around which DNA is wrapped. Each histone core is composed of two copies of each of the histone proteins H2A, H2B, H3 and H4. Nucleosome not only plays a structural role, but also participates in regulating transcription through its positioning [1], [2], [3], [4]. Nucleosomes are regularly arranged around the transcriptional start site (TSS) of protein-coding genes and regulate the accessibility of regulatory elements for controlling transcription. Nucleosomes show depleted at the promoters of the genes showing actively-transcribed genes, in order to expose DNA segments for the binding of transcriptional factors. In the interior of genes, nucleosomes strongly prefer to occupy exon starts, suggesting a potential role in splicing [5], [6]. These noticeable organizational patterns provide clues into mechanistic principles of nucleosome-related gene regulations.

Recently, we have described the variation of nucleosome-space occupancy (NO) density as an important feature of gene-expression regulation in the mouse embryonic stem cell (ESC) based on a survey that partitions genomic sequences into nucleosome-rich and nucleosome-poor gene islands. These clustered genes show clear associations with DNA composition, transcription, and several epigenetic mechanisms [7]. To further understand the role of NO variations in controlling transcriptional activity, we generated a genome-wide NO map in the mouse cerebrum and testis based on a ChIP (H3)-seq protocol (SOLiD sequencing [8] and profiled the two transcriptomes at the same time. In this paper, we mainly performed comparative analysis of NO density between cerebrum and testis in the mouse, and correlated the density of NO distribution to genomic transcriptional activity. We further supported that nucleosome enrichment or depletion occurred within a relative larger genomic region could play a role in regulating gene expressions. Moreover, we described the distinctive binding levels of nucleosomes between HK and TS genes.

## Materials and Methods

### Data sources

We acquired cerebrum and testis samples from 10-week old male BALB/c mouse and carried out rmRNA experiments as described previously [9]. We performed ChIP-seq experiments according to a published protocol [10], tissues were homogenized and fixed with 1% formaldehyde, and then fragmented to a size range of 200 to 1,000 bases. Solubilized chromatin was incubated at 4°C overnight with antibody against histone H3 (Abcam, #AB1791). After cross-link reversaland Proteinase K treatment, DNA samples wereextracted with phenol-chloroform, precipitated under ethanol, treated with RNase and Calf Intestinal Alkaline Phosphatase, and purified with a MinElute Kit (Qiagen). Sequencing libraries were generated from about 10 ng of ChIP DNA by adaptor ligation, gel purification and 13 cycles of PCR. We obtained sequence tags using SOLiD system (Applied Biosystems Inc) according to the manufacturer's specifications. The ChIP-seq and rmRNA-seq data have been submitted to NCBI SRA with accession code SRA010955. The handling of mice and experimental procedures were guided and approved by Beijing Municipal Science & Technology Commission with SYXK2009-0022.

### Data analysis

We mapped the sequence reads to the mouse genome (mm9) using a custom-designed SOLiD mapping pipeline and aligned the sequences by allowing up to five mismatches out of 50-bp reads. We retrieved public RNA-seq and H3 ChIP-seq data for the mouse stem cell, nucleosome binding data for the human resting CD4+ T cell [7], [10], [11] from NCBI (Table S1).

We use RefSeq known genes for all analyses. If a gene has several isoforms, we take the one that has more exons, yielding 19,043 RefSeq known genes. We classified the selected genes into HCP, LCP, and ICP genes based on their promoter categories [12]. If at least five successive genes (based on their genomic coordinates) are in the same promoter category (HCP, ICP, or LCP), these genes are considered as clustered. We mapped sequence reads generated from rmRNA-seq (ribosomal RNA-minus) and H3 ChIP-seq protocols to these genes to obtain expression and NO information. We normalized the read counts based on the gene length and the number of unique reads from each library.

We divided chromosome into a sliding window of 100 kb in length and counted the number of reads from RNA-seq and H3 ChIP-seq protocols for each window after normalizations. We used the normalized signals as indicators for transcriptional activity and NO intensity [7]. We performed Pearson Correlation Test (P<0.01) to evaluate the correlation of NO distribution among mouse stem cell, cerebrum, and testis. Meanwhile, we also detected some genomic regions have differential NO between mouse cerebrum and testis by Fisher-exact Test (P<1e-5) built in the IDEG6 software [13]. We used K-means clustering strategy to find genomic regions that share similar NO intensity and transcription activity. As a result, all the genomic regions were classified into two gene groups (LOG and HOG). We performed statistical analyses and plotted the results with the R software (Version 2.8.0) [14].

We categorized genes into those of LOG and HOG and aligned their transcript-centric positions—TSS and TTS—in a ±1-kb window. We counted tags in a 5-bp window and plotted the normalized tag counts based on the sequence of transcription units.

## Results

### Consistent pattern of NO distribution in the mouse tissues

**Figure 1:**
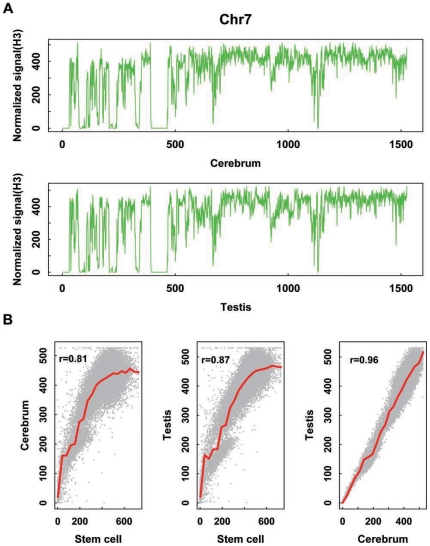
NO Profiling of mouse chromosome 7 based on correlation analysis on cerebrum and testis. We show here (A) tag density based on ChIP (H3)-seq data at a 100-kb window (see also Figure S3) and (B) correlation of NO intensity at a 100-kb chromosome window (P<0.01) among mouse cerebrum, testis, and stem cell.

We firstly acquired 25 and 28 million uniquely mapped chip-seq (H3) reads from the two tissues and plotted the signals of NO density along chromosomes in a 100-kb sliding window based on sequence read counts. Similar to our previous finding in mouse ESCs [7], we are able to clearly define nucleosome-rich and nucleosome-poor regions (Figure 1A), and identify a high degree of similarity in NO distribution between the cerebrum and testis (Figure 1B). This result indicates that there is a nearly consistent NO distribution in the mouse tissues. Furthermore, we have confirmed the correlation between NO density and GC composition (Figure S1) to demonstrate that nucleosome positioning is related to the composition dynamics of local sequences [15], [16], [17].

### The effect of density of NO distribution on distinctive expression of HK and TS genes

**Figure 2:**
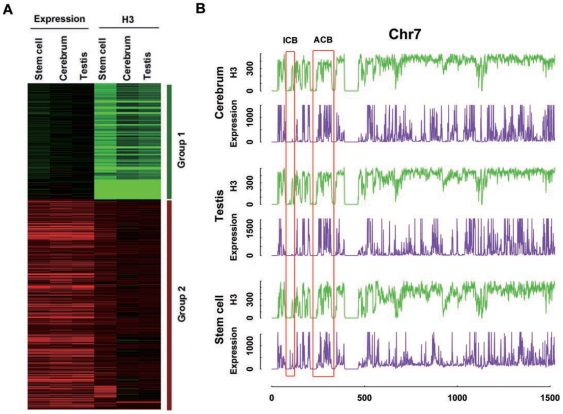
Clustering of nucleosomal and transcriptional features (A) and an example to illustrate actively-transcribed chromosomal blocks (ACB) and inactively-transcribed chromosomal blocks (ICB) (B). The NO signals and sequence tags from rmRNA-seq are aligned in a 100-kb window (the tag densities from high to low are scaled from red to green). Criteria about the grouping scheme (Group1 and Group2) are discussed in the main text and they are corresponding to LOG and HOG. We observed large genomic regions where NO intensity and transcriptional activity are constantly low (ICB) or high (ACB), and examples are highlighted in red boxes.

To further exploit the association between NO density and transcriptional activity, we acquired the profiles of the genomic transcriptional activity in the mouse cerebrum and testis using the RNA-seq method. Based on these newly-obtained data, coupled with the publicly available data from the mouse ESCs [7], [10], we firstly carried out a k-means clustering [14] analysis between the density of NO distribution and transcriptional activity at the whole genomic level among three tissues, using the number of reads at a 100-kb window, and found that the whole genome was divided into two different groups based on the distinctive features of nucleosome density and transcriptional activity(Figure 2A). The Group1 genomic regions have relatively poor nucleosome coverage and significantly lower expression as compared to the opposite trend of the Group2 regions; we termed these two groups as high and low (nucleosome-space) occupancy groups or HOG and LOG, respectively. This clustering result was also evident from comparative plotting the signals of NO density and transcriptional activity along each chromosome. We are able to define actively-transcribed chromosomal blocks (ACB) and inactively-transcribed chromosomal blocks (ICB; [4], [10], [18], where nucleosomes are either relatively enriched or depleted. These ACB and ICB show correlations with nucleosome-rich and nucleosome-poor gene islands, and such a correlation appears conserved across tissues and cells (Figure 2B).

**Figure 3:**
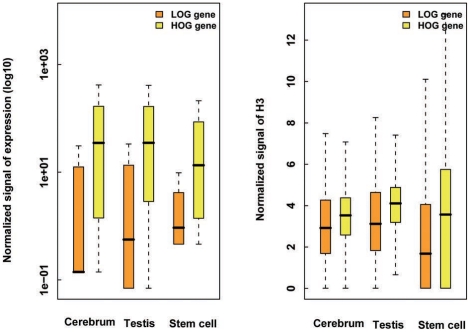
Box plots showing differential expression and NO levels between LOG and HOG genes.

Corresponding to the LOG and HOG genomic regions, the mouse protein-coding genes can be categorized as the LOG genes and HOG genes, the number of which was respectively counted as 3,939 and 15,004. The LOG genes tend to be harbored by nucleosome-poor chromosomal regions, where the gene expression level is also rather low; the HOG genes, however, show the opposite trend (Figure 3). Gene ontology and promoter sequence analyses [12], [19] on the LOG genes yielded significant enrichment of the pathways concerning cell communication and system process (Table S2). Within these two categories, a majority of the genes are assigned into certain functional sub-categories, such as signaling transduction and stimulus response pathways. Some of these genes are tissue-specific and involved in development and mature of tissues or organs. Obviously, these genes are mainly involved in tissue-specific functions and temporal regulations in response to stimulus [20]. Furthermore, we were able to partition the promoter sequence of the 3,939 LOG genes into 2,263 LCP (low CpG promoter), 700 ICP (intermediate CpG promoter), and 976 HCP (high CpG promoter) genes, where LCPs tend to be associated with tissue-specific (TS) genes [10], [21]. In contrast, most of the HOG genes—among them 10,783 possess HCP—are house-keeping (HK) genes and involved in metabolic process, biological process, and cellular component (Table S2). Moreover, we investigated the gene expression breath [21], [22] for LOG and HOG genes among 19 mouse tissues/cells based on the newly-generated (JY and SNH unpublished data) and publicly available RNA-seq data (Table S3) and found that only 13% LOG genes were found expressed in 18 out of 19 tissues whereas most LOG genes exhibited tissue-specific expression. In contrast, most HOG genes (56%) showed universal expression in 18 out of 19 tissues (Figure S2). We concluded that TS and HK genes appear to have distinct NO and expression patterns; TS genes fall into NO-poor chromosomal regions and show low expression level as compared to the opposite trends for the same features of HK genes. These analyses indicate that the NO density along chromosome shows well positively correlated to the distinctive transcriptional activities of the HK and TS genomic regions, which further implied that nucleosomes enrichment or depletion within larger genomic regions could as an epigenetic regulator for gene expression.

### Differences in the NO density associated with tissue-specific expression

**Figure 4:**
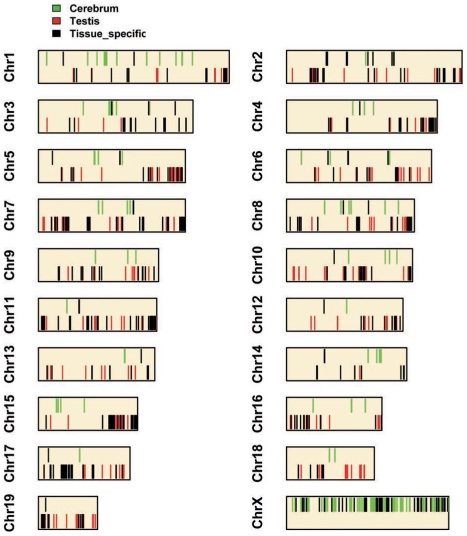
Profiles of tissue-specific and NO-intensive regions over each chromosome. Genomic regions showing higher NO intensity in mouse cerebrum (red bars, lower panel) and testis (green bars, upper panel) are plotted. Some of these regions that harbor cerebrum- and testis-specific genes are highlighted in black. There is no information for Y.

Although the NO distribution is rather similar between cerebrum and testis in the mouse, we can still find about 6% (P<1e-5) genomic regions where NO signals or intensity is significantly variable (Figure 4). Importantly, these genomic regions are organized as clusters or largely continuous genomic regions and are well associated with tissue-specific expression. In the cerebrum, we detected 864 100-kb genomic regions as NO-intensive when compared to the corresponding genomic regions of the testis and found that most of them are organized as clusters or concatenated as continuously genomic regions. Further scrutiny shows that there are 244 protein-coding genes within these genomic regions, which show constantly nucleosome-enriched compared to those in testis. Surprisingly, in the mouse cerebrum, chromosome X shows a broadly elevated level of NO, where the genes encoded for transporters of both macromolecules and small molecules, and ion-binding proteins are enriched, and some of these genes have been reported that there could be important functions in cerebrum [19], [23], [24]. Furthermore, through comparing gene expression analysis between the mouse cerebrum and testis, we identified that (72%) of these nucleosome-enriched genes in the cerebrum have higher transcriptional activities than those of the same genes expressed in the testis. Similarly, in the testis, we found 725 genomic regions covering (2199) protein-coding genes that harbor more nucleosomes as compared to those of cerebrum. Moreover, most (52%) of these genes show significantly higher transcriptional activities in the testis compared to those in the cerebrum. These analyses suggested that the change of NO density between cerebrum and testis can be well correlated to the tissue-specific expression. This further implied the role of nucleosome density in regulating gene expression.

### How does the change of NO density regulate gene expression?

Above analyses suggested the functional role of the change of NO density in regulating gene expression. But how does this nucleosome organization regulate gene expression? We here described two views based on present results. Firstly, the global investigation of the NO distribution exhibited that nucleosomes enrichment or depletion is occurred at a relatively larger genomic region, which normally covers multiple genes. We surveyed the distribution of HOG and LOG genes in the mouse genome and found that most of the genes of the two groups, including 85%of LOG genes and 87% of HOG genes, are organized as gene clusters (>5 genes each) where nucleosomes are constantly either depleted or enriched, respectively. In fact, these gene clusters are also associated with larger nucleosome-rich or nucleosome-poor, and ACB or ICB (Figure S3). This result suggests that the regulation of NO density variations engages larger genome regions or gene clusters and is not limited to individual genes. Therefore, based on present knowledge that the change of transcriptional activity at larger genome regions could be generally suffer from the regulation of chromatin structure [25], [26], we considered that this regulation of NO density could directly impact on chromatin structure, but at present, we have no any experiments evidence for proving this possibility.

**Figure 5:**
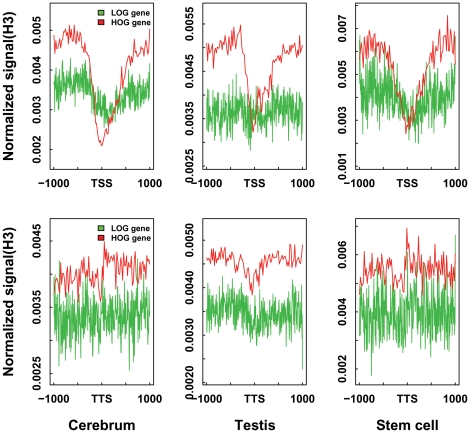
Nucleosome positioning between LOG and HOG genes. Normalized nucleosome signals over transcription start sites (TSS; top row) and transcription termination sites (TTS; bottom row) of nucleosome LOG and HOG genes are displayed.

In addition, we cannot exclude other effects of NO density variations on gene expression and regulation. In the case of LOG genes, the lowly-expressed TS genes, often found in NO-poor regions, tend to lose nucleosome positioning signal at TSS and TTS as compared to those of HOG genes that show the opposite trends (Figure 5). Since the regular nucleosome positioning around genes is believed to regulate gene expression [3], we suggest that the LOG genes may only be loosely regulated by nucleosome binding.

In this study, we also extended our analyses to the human data based on a public dataset [11], where we not only found similar NO variations but also identified NO-poor or NO-rich genomic regions as well as their gene clusters in a context of LCP or HCPs (Figure S4). The results validated our findings in the mouse tissues and suggest that the regulatory mechanisms for NO variation are rather universal and conserved across mammalian lineage.

## Discussion

This study is focused on how nucleosomes are organized locally across the relative larger genomic regions or gene clusters beyond individual transcriptional units, and its effects on gene expression. Firstly, we acquired the profiles of NO density and transcriptional activity along chromosomes in the mouse cerebrum and testis based on the Chip(H3)-seq and RNA-seq protocols. Through comparative analyses of NO density and transcriptional activity between the cerebrum and testis, we identified a consistent distribution of NO density in the mouse cerebrum, testis and ESCs, and found that NO density is well associated with distinctive expression levels between HK and TS genes. This conclusion suggests that the change of NO density should have a functional role in regulating gene expression. Although NO profiles show highly agreed in the mouse tissues, we still can identified some subtle differences in NO profiles between the cerebrum and testis, and further found that these variable NO profiles are well associated with the tissue-specific expression pattern.

The previous papers revealed that the relationships between the nuclesome position and gene expression. In the yeast [27], [28], [29], C. elegant [30], fruit fly [31], mouse [10], [32] and human [11], the nuclesome occupancy in promoters is negatively related with gene expression, which is ready for transcription start. In Plasmodium falciparum, the relationship is inconspicuous caused by the extreme AT content (∼80%) in genome [33], [34]. However, these studies only focused on the dynamics of nucleosome binding around the promoter regions. Our results are derived from the survey at relative large genomic regions, not limiting to promoter regions, but including the gene body regions. We believed that NO dynamics at large levels could be linked to the status of chromosome structures, open or close, and thus indirectly regulate gene expression, although there is no exact mechanism for explaining it. Currently, we considered that NO dynamics at large genomic regions may be used for establishing the required environment for other extensively and effectively regulation system, such as histone modifications or protein recognition. In fact, recent some findings also provided the potential regulatory roles of NO density. Firstly, NO density shows distinct between exons and introns, which has been suggested to be associated with splicing process [35], [36]. Secondly, in human cells, DNA at promoters, enhancers, and TFBSs generally followed high NO [37], which suggested the roles in regulating gene expression. Another research also provide evidence that GC-depended NO attract the p53 protein in human cells [38]. Additionally, our previous study performed in mouse stem cell have suggested that there are good correlations between NO density and histone modifications at large genomic levels [39], which can support our hypothesis.

A frequently-asked question is whether we can use ChIP experiment to evaluate nucleosome occupancy in this analysis. Since immunoprecipatation efficiency can depend on epitope accessibility, some histones that are bound by large protein complex might be more inaccessible for H3 antibody [27], and thus the associated DNA segments might be depleted in the H3 ChIP experiment. This concern is very reasonable. However, we have some reasons to say that this affect may be subtle. Firstly, when using the data based on the MNase digestion that makes double-strand DNA cuts between nucleosomes and shows a distinct experimental principle from mmunoprecipatation to analyze NO in human T cells, we obtained a similar NO pattern in human genomes to that in mouse genome. This constant result from two independent experiments suggests that ChIP could have a weaker influence on the final result. Additionally, we cited a published paper [10] to further prove this opinion. In the paper, they generated the ChIP-seq reads of pan-H3, H3K4me3, H3K27me3 and the sequencing reads from unriched whole-cell extract DNAs (as a control). When evaluated the obtained ChIP-data, they compared cumulative distributions of the observed and randomized reads densities (averaged over 1-kb windows) across the mouse genome. The observed distributions for pan-H3 ChIP and whole-cell extract are virtually identical to the randomized distributions, indicating that ChIP-Seq generates unbiased data from unenriched samples. In contrast, the observed distributions for H3K4me3 and H3K27me3 enriched samples show clear excess of extreme values. We believed that the consistent pattern between pan-H3 ChIP and whole-cell extract can also indicate that the bias of ChIP experiment have a subtle influence on the sampling of DNA segments.

## Supporting Information

Figure S1 **Box plots showing the relationship between DNA composition and NO intensity.** There is a significant positive correlation between GC content and the NO intensity in the mouse cerebrum, testis, and stem cell.
(PDF)

Figure S2 **LOG and HOG genes are plotted as a function of expression breadth.** The fractions of LOG and HOG genes are plotted against expression breadths. Majority of the widely expressed genes are HOG genes; on the contrary, most of the tissue-specific genes are LOG genes.
(PDF)

Figure S3 **Profiles of transcription activity and NO intensity in mouse cerebrum, testis, and stem cell.** For each chromosome, the first row on the top of each chromosome indicates the profile of nucleosome density, which was estimated based on the number of tags in a 100-kb window after normaliztion. The second row indicates the profile of the transcriptomes. The third row indicates the density of clustered genes on the two strands. Clustered genes are defined as a set of five or more neighboring genes in the same promoter group (HCP, LCP, and ICP).
(PDF)

Figure S4 **NO profiles of human chromosome 11.** The red boxes highlight LCP-gene clusters where nucleosomes are scarce
(PDF)

Table S1 **The number of unique reads from libraries.**
(DOC)

Table S2 **GO analysis of LOG and HOG genes.**
(XLS)

Table S3 **A list of tissues or cells.**
(DOC)
