# Supplementary figures and images for: The Disequilibrium of Nucleosomes Distribution along Chromosomes Plays a Functional and Evolutionarily Role in Regulating Gene Expression
Source: PLoS One. 2011 Aug 19;6(8):e23219. doi: 10.1371/journal.pone.0023219 (PMC3158759; doi:10.1371/journal.pone.0023219)

**Stem cell**

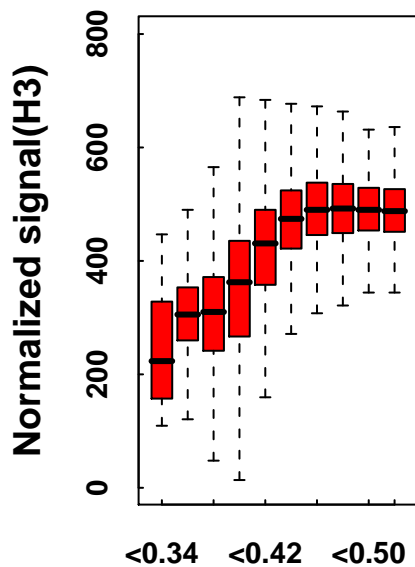

**GC content**

**Cerebrum**

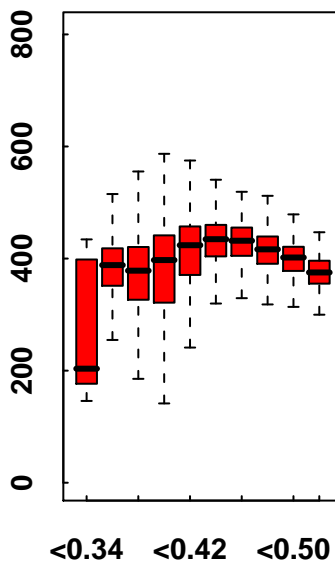

**GC content**

**Testis**

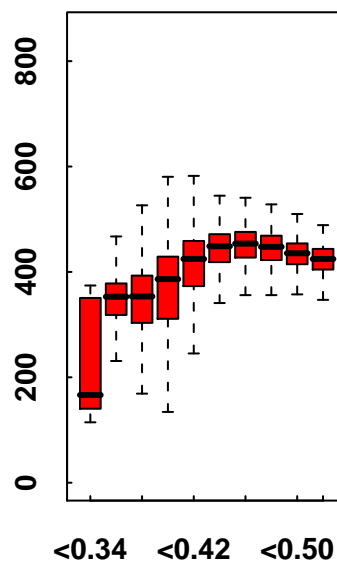

**GC content**

Supplement: Figure S1 — Box plots showing the relationship between DNA composition and NO intensity. There is a significant positive correlation between GC content and the NO intensity in the mouse cerebrum, testis, and stem cell. (PDF) [file pone.0023219.s001.pdf]

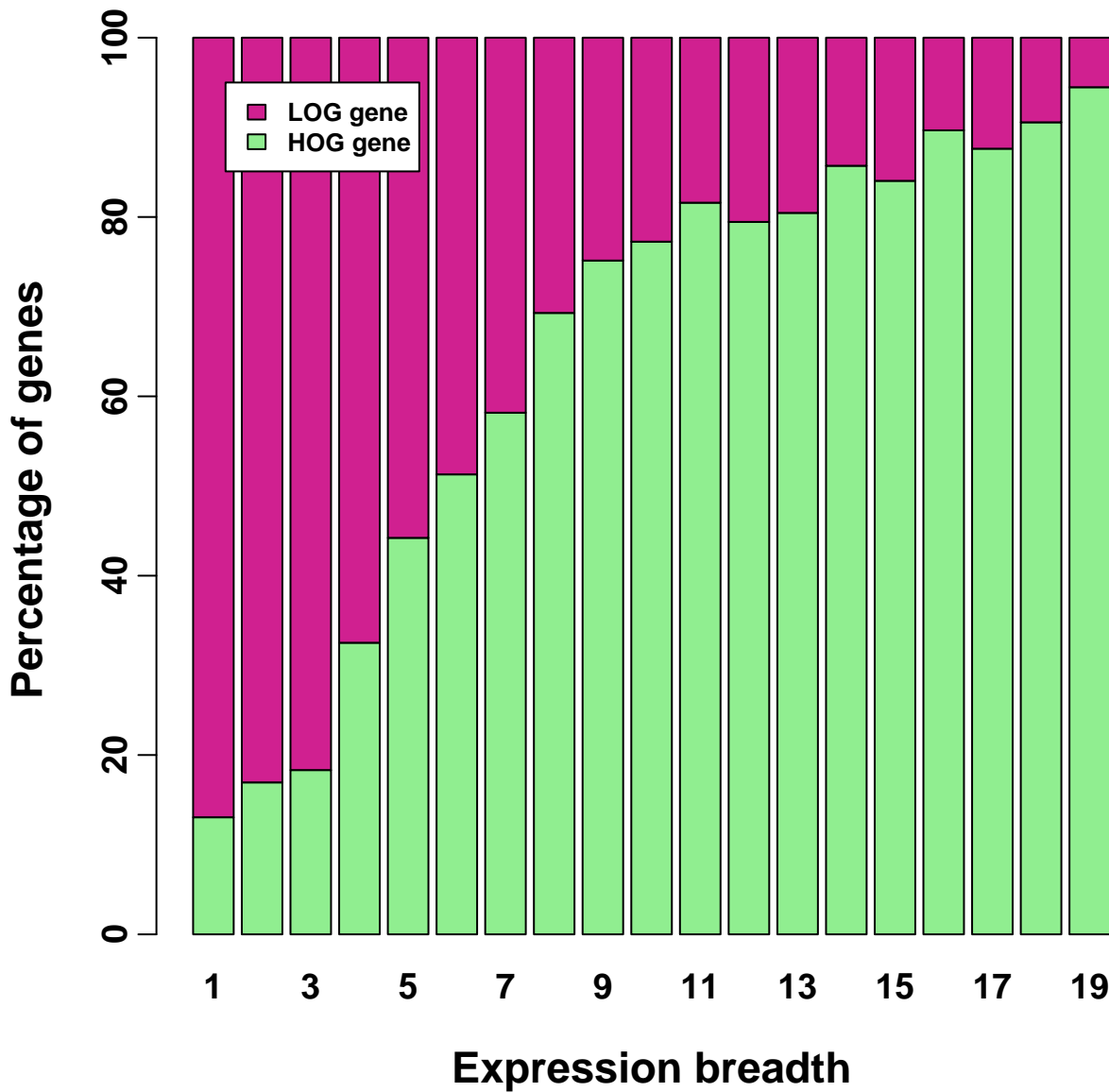

Supplement: Figure S2 — LOG and HOG genes are plotted as a function of expression breadth. The fractions of LOG and HOG genes are plotted against expression breadths. Majority of the widely expressed genes are HOG genes; on the contrary, most of the tissue-specific genes are LOG genes. (PDF) [file pone.0023219.s002.pdf]

# Chr11

Normalized signal(H3)

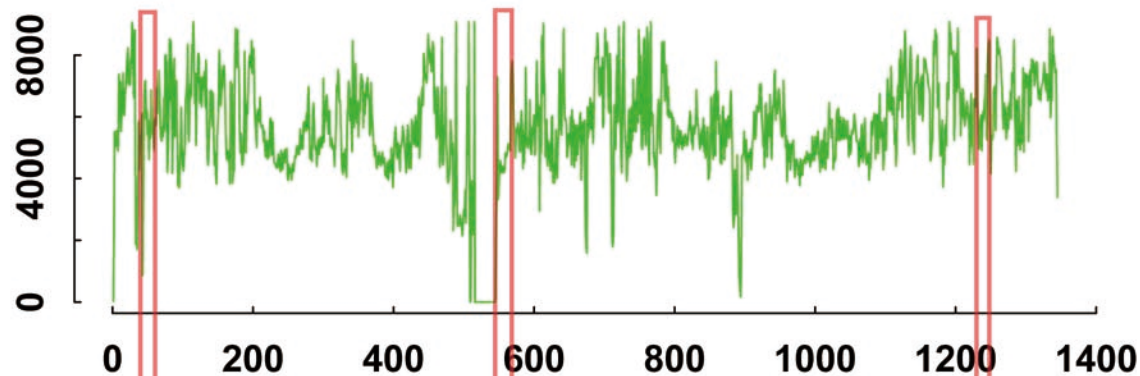

GC content

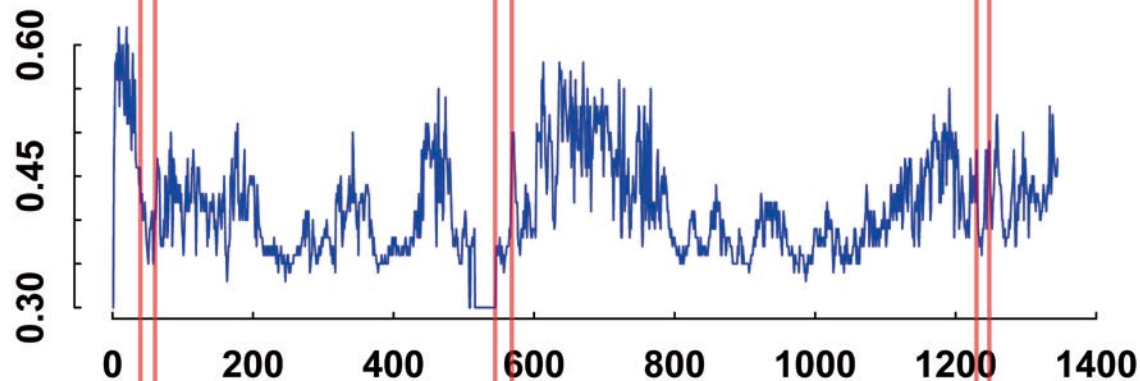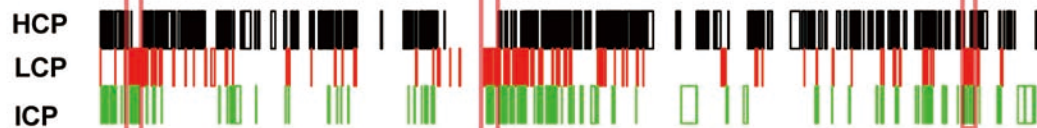

Gene density

Supplement: Figure S4 — NO profiles of human chromosome 11. The red boxes highlight LCP-gene clusters where nucleosomes are scarce (PDF) [file pone.0023219.s004.pdf]
