# Supplementary material for: The Disequilibrium of Nucleosomes Distribution along Chromosomes Plays a Functional and Evolutionarily Role in Regulating Gene Expression
Source: PLoS One. 2011 Aug 19;6(8):e23219. doi: 10.1371/journal.pone.0023219 (PMC3158759; doi:10.1371/journal.pone.0023219)
Supplement: Figure S3 — Profiles of transcription activity and NO intensity in mouse cerebrum, testis, and stem cell. For each chromosome, the first row on the top of each chromosome indicates the profile of nucleosome density, which was estimated based on the number of tags in a 100-kb window after normaliztion. The second row indicates the profile of the transcriptomes. The third row indicates the density of clustered genes on the two strands. Clustered genes are defined as a set of five or more neighboring genes in the same promoter group (HCP, LCP, and ICP). (PDF) [file pone.0023219.s003.pdf]

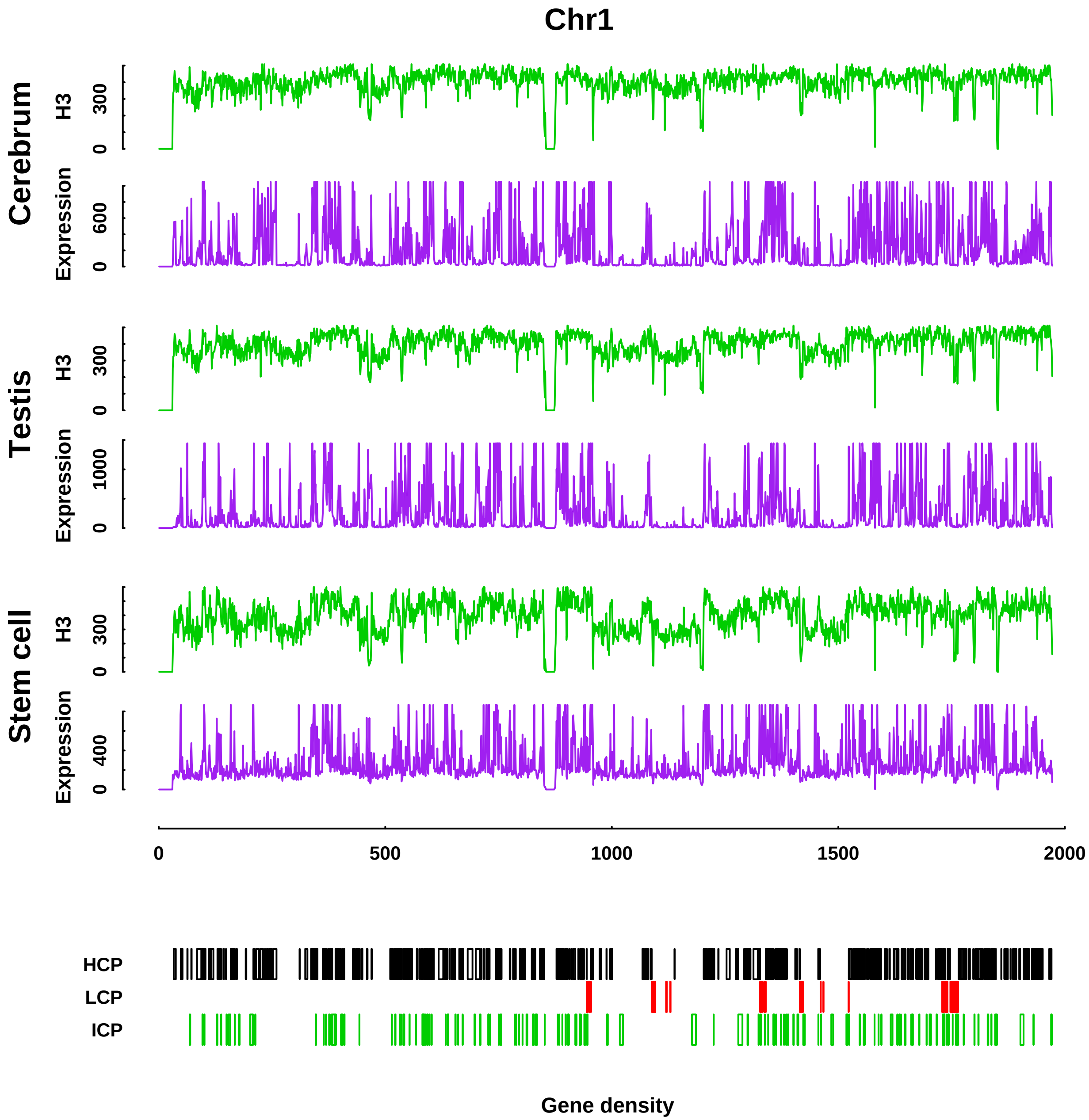

Chr1
Cerebrum
H3
0
300
Expression
0
600
Testis
H3
0
300
Expression
0
1000
Stem cell
H3
0
300
Expression
0
400
0
500
1000
1500
2000
HCP
LCP
ICP
Gene density

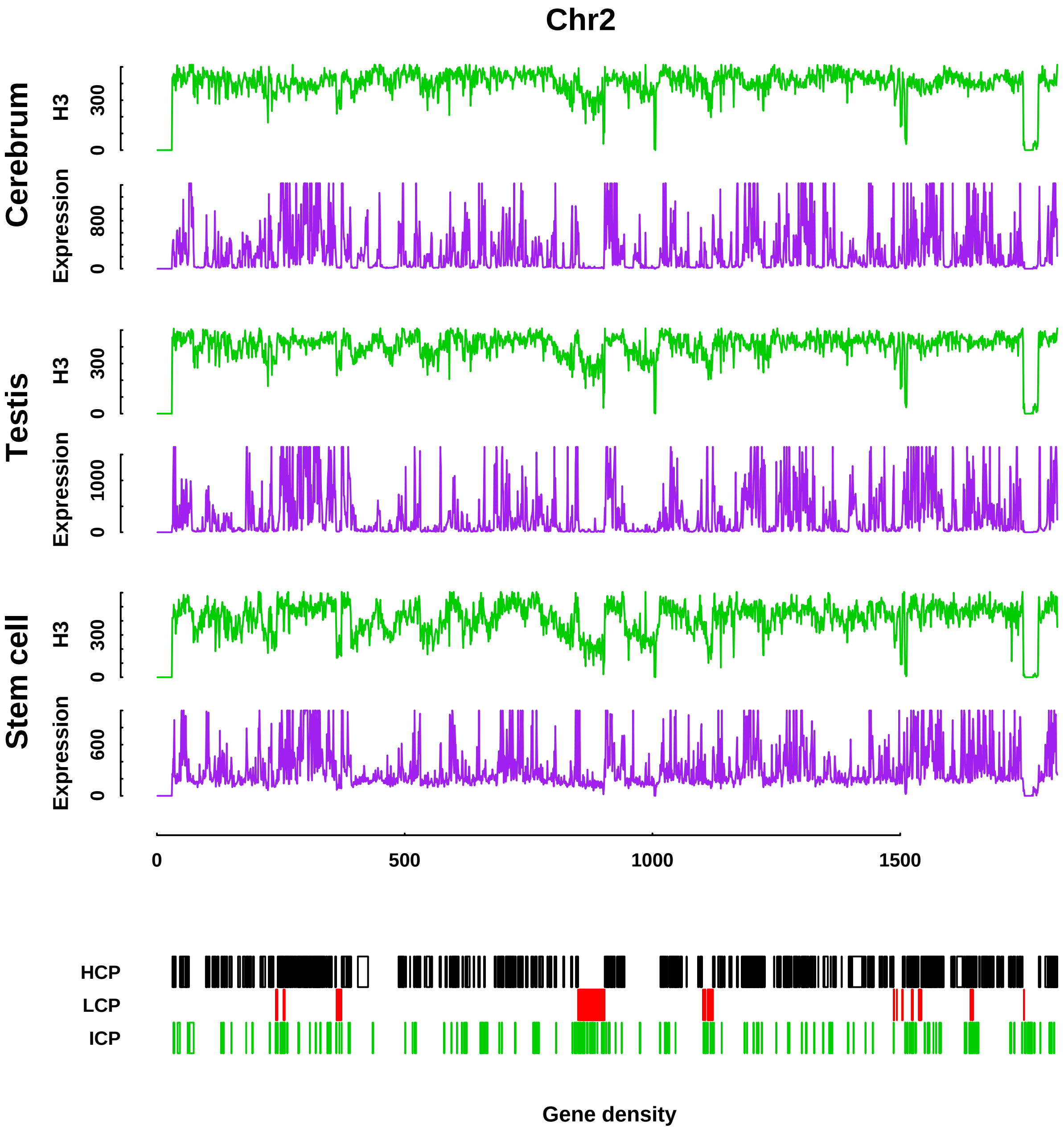

Chr2
Cerebrum
H3
0
300
Expression
0
800
Testis
H3
0
300
Expression
0
1000
Stem cell
H3
0
300
Expression
0
600
0
500
1000
1500
HCP
LCP
ICP
Gene density

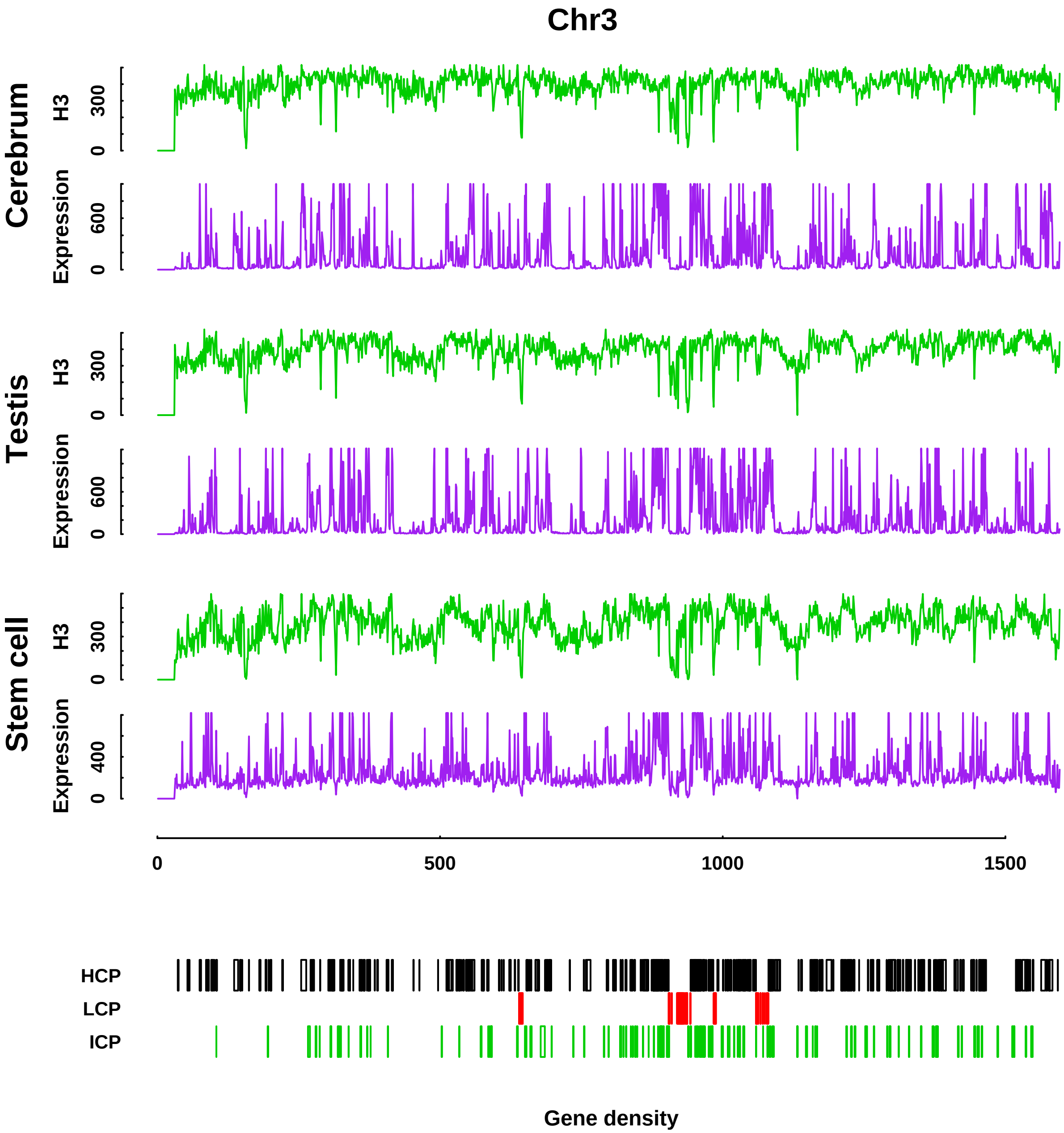

Chr3
Cerebrum
Testis
Stem cell
H3
Expression
0
300
600
400
0
500
1000
1500
HCP
LCP
ICP
Gene density

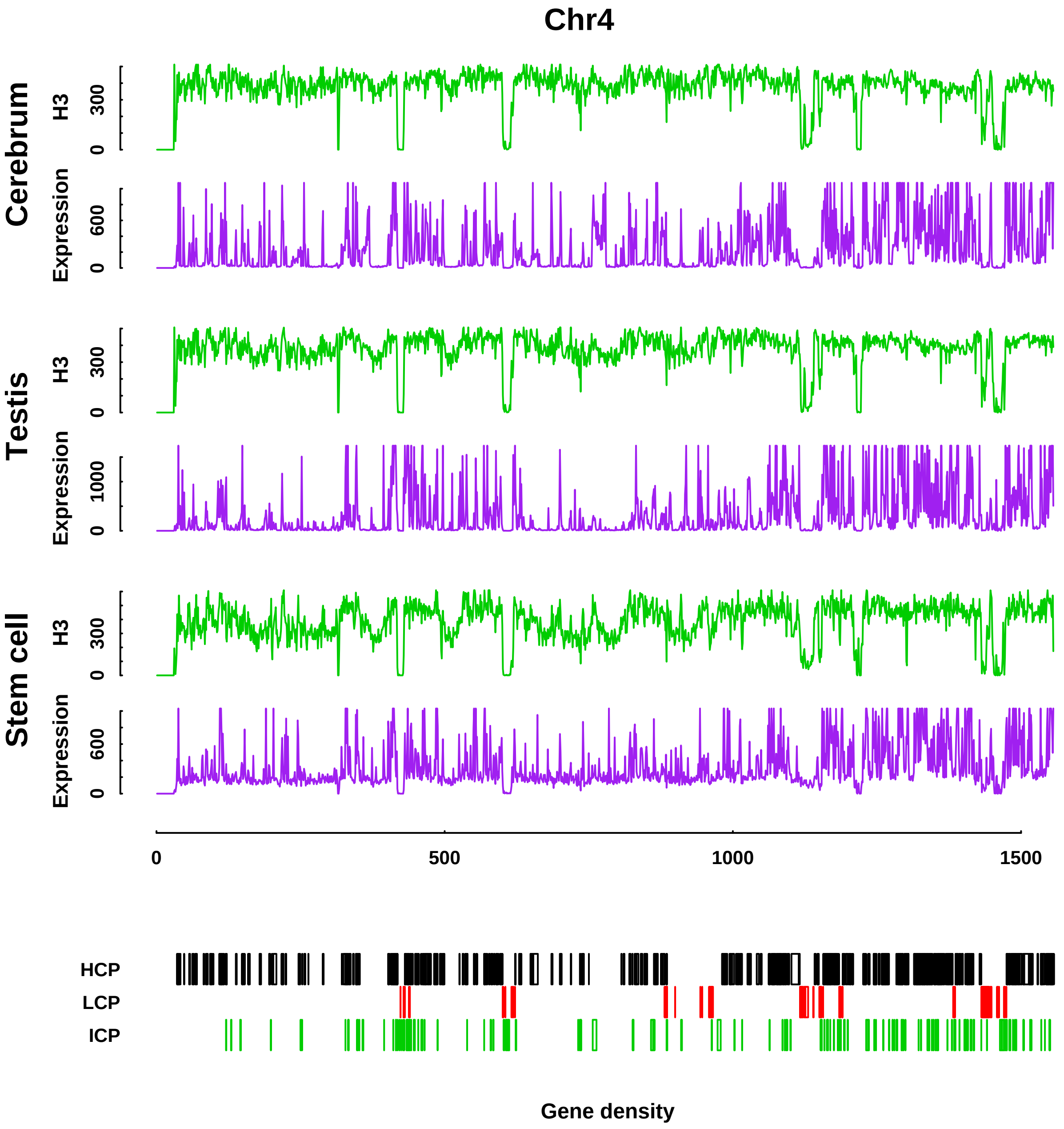

Chr4
Cerebrum
H3
0
300
Expression
0
600
Testis
H3
0
300
Expression
0
1000
Stem cell
H3
0
300
Expression
0
600
0
500
1000
1500
HCP
LCP
ICP
Gene density

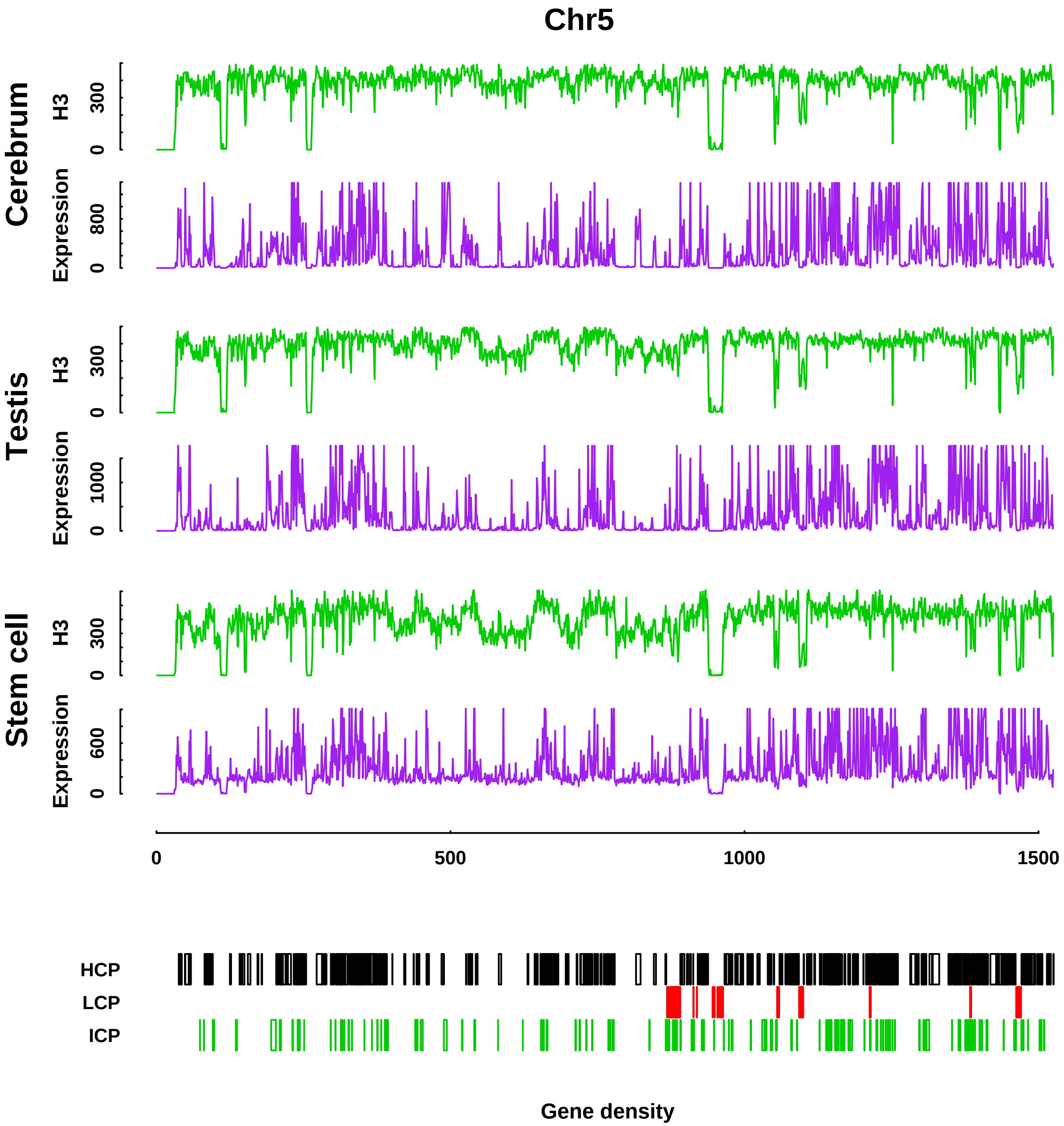
Chr5
Cerebrum
H3
0
300
Expression
0
800
Testis
H3
0
300
Expression
0
1000
Stem cell
H3
0
300
Expression
0
600
0
500
1000
1500
HCP
LCP
ICP
Gene density

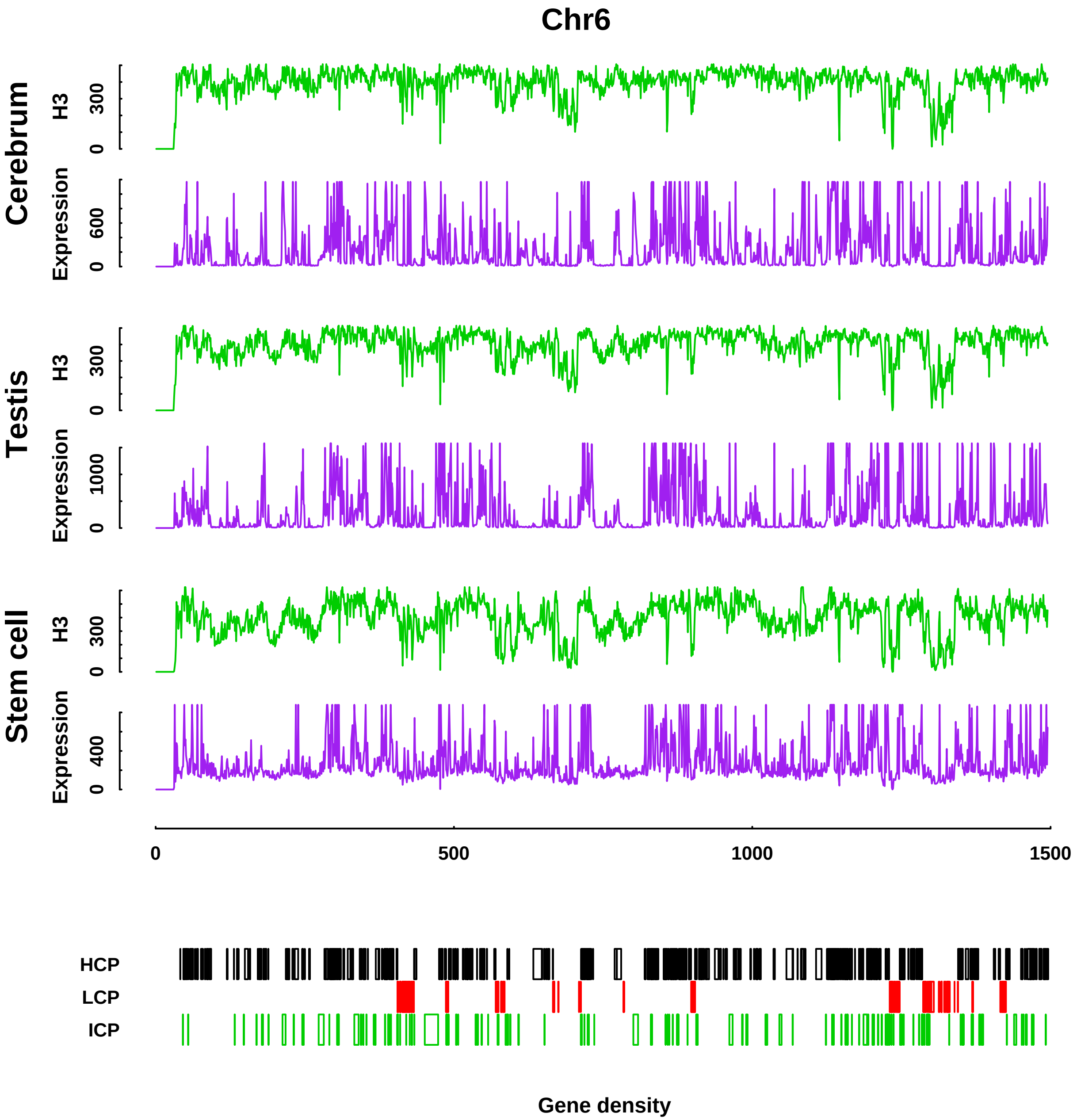
Chr6
Cerebrum
H3
0
300
Expression
0
600
Testis
H3
0
300
Expression
0
1000
Stem cell
H3
0
300
Expression
0
400
0
500
1000
1500
HCP
LCP
ICP
Gene density

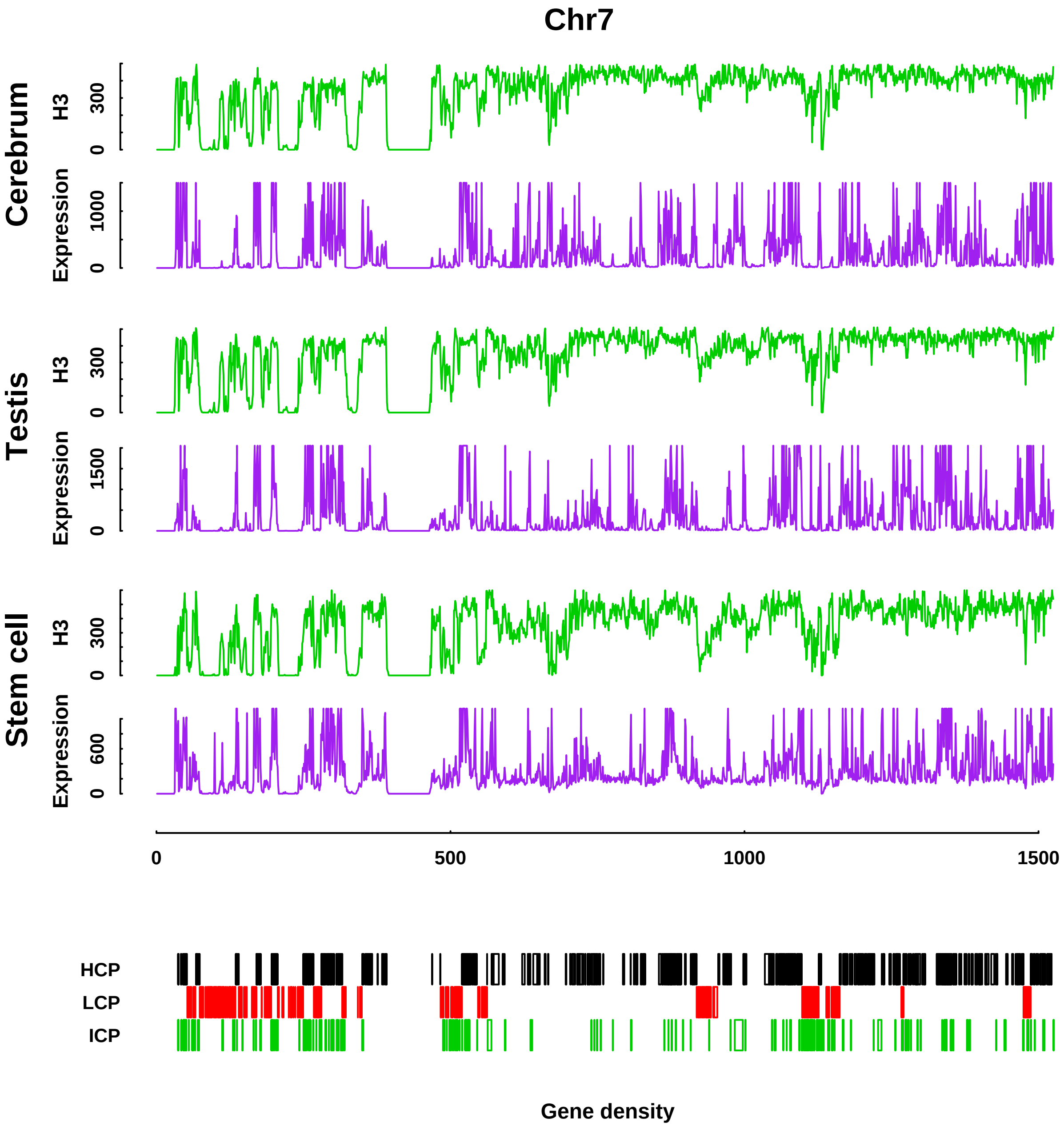

Chr7
Cerebrum
H3
0
300
Expression
0
1000
Testis
H3
0
300
Expression
0
1500
Stem cell
H3
0
300
Expression
0
600
0
500
1000
1500
HCP
LCP
ICP
Gene density

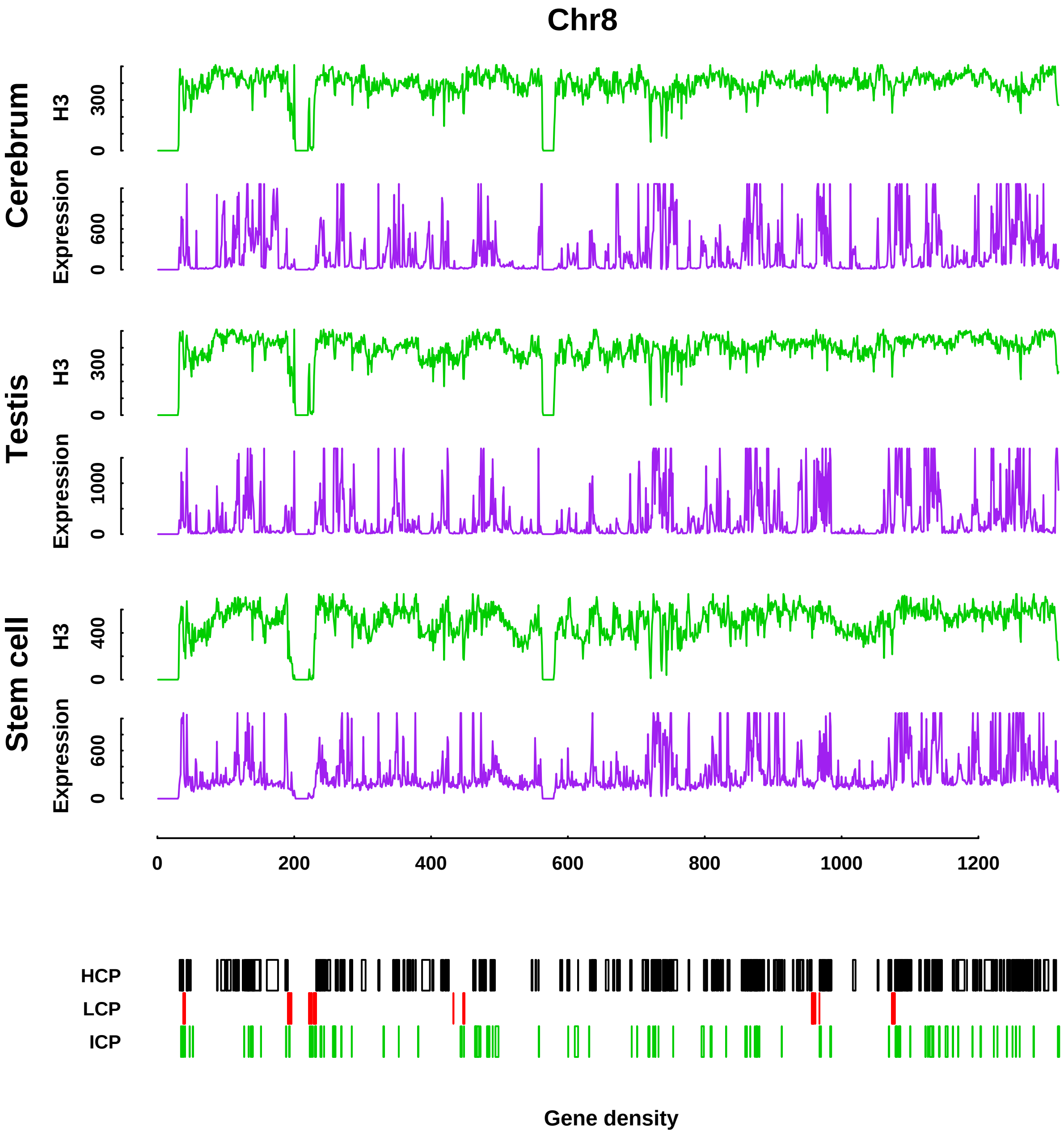

Chr8
Cerebrum
H3
0
300
Expression
0
600
Testis
H3
0
300
Expression
0
1000
Stem cell
H3
0
400
Expression
0
600
0
200
400
600
800
1000
1200
HCP
LCP
ICP
Gene density

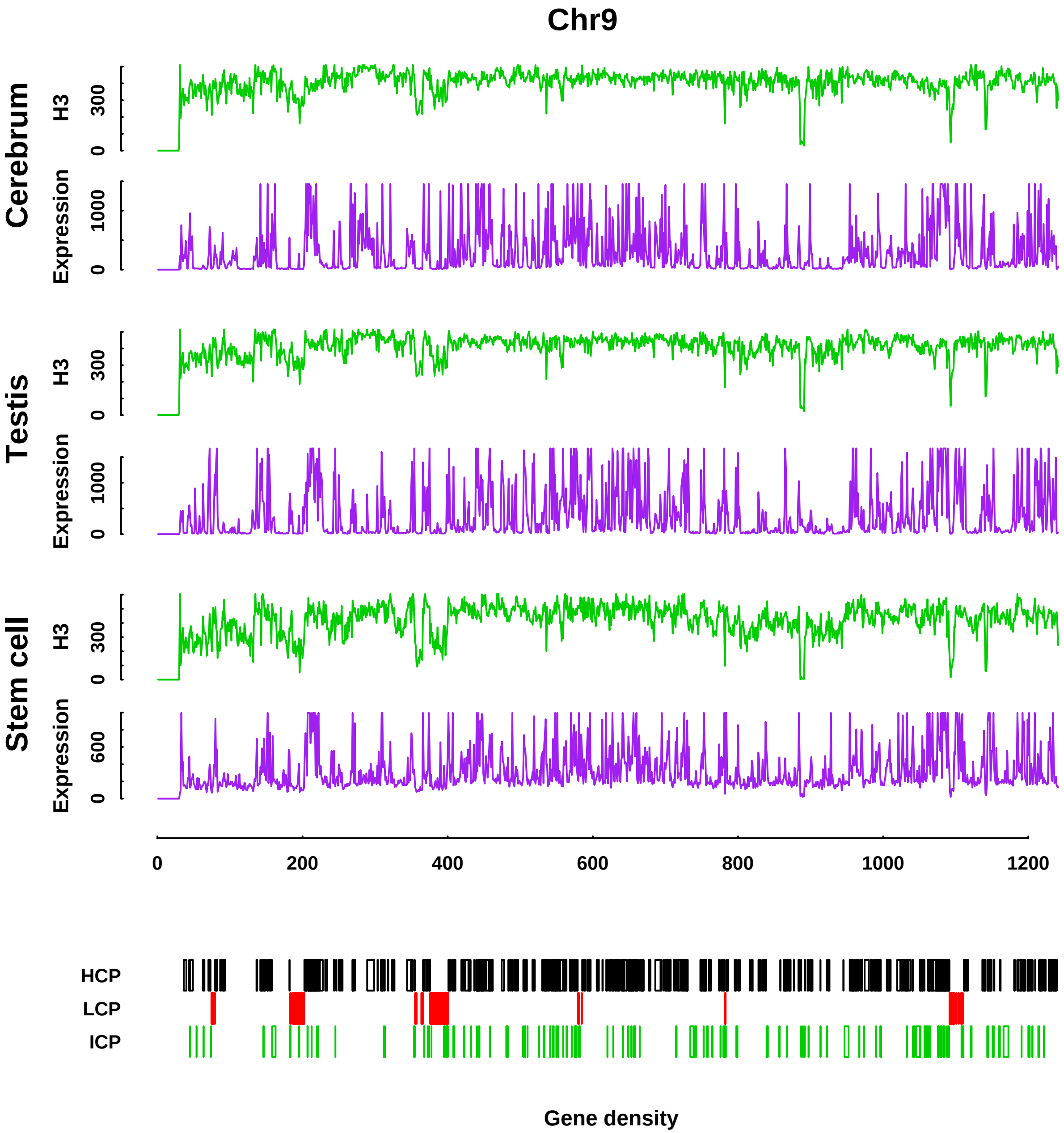

Chr9
Cerebrum
H3
0
300
Expression
0
1000
Testis
H3
0
300
Expression
0
1000
Stem cell
H3
0
300
Expression
0
600
0
200
400
600
800
1000
1200
HCP
LCP
ICP
Gene density

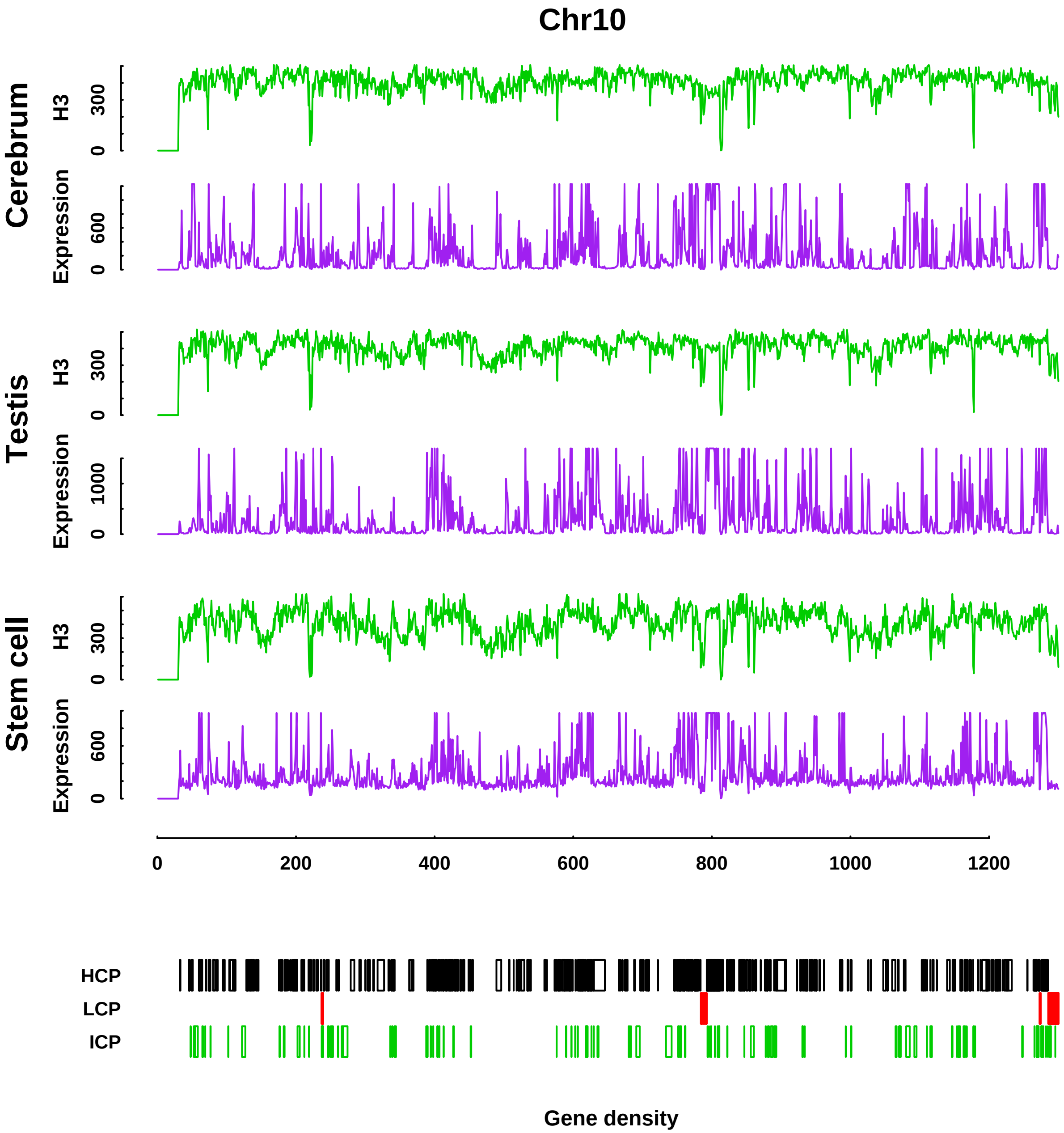
Chr10
Cerebrum
H3
0
300
Expression
0
600
Testis
H3
0
300
Expression
0
1000
Stem cell
H3
0
300
Expression
0
600
0
200
400
600
800
1000
1200
HCP
LCP
ICP
Gene density

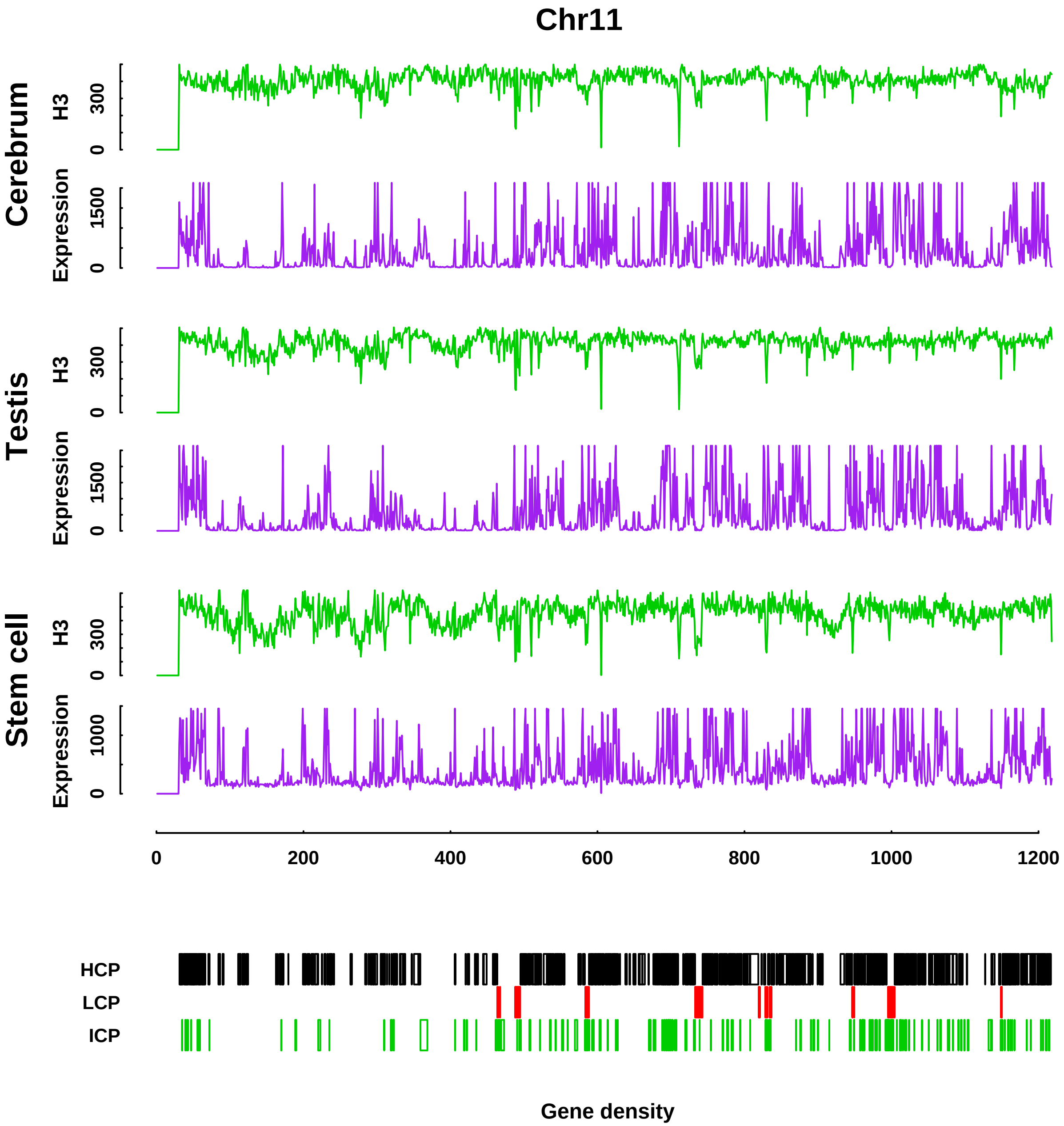
Chr11
Cerebrum
Testis
Stem cell
H3
Expression
0
300
1500
1000
0
200
400
600
800
1000
1200
HCP
LCP
ICP
Gene density

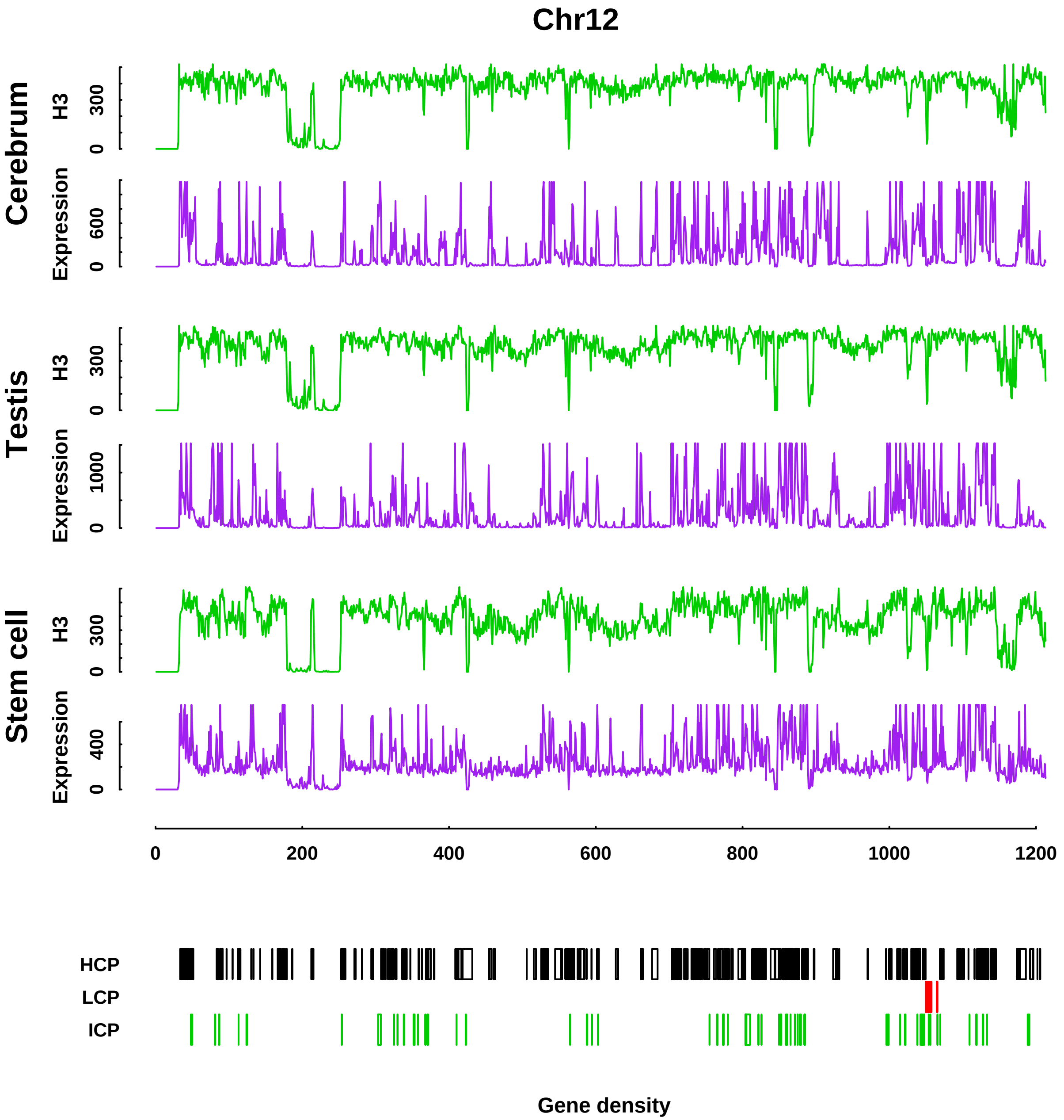

Chr12
Cerebrum
H3
0
300
Expression
0
600
Testis
H3
0
300
Expression
0
1000
Stem cell
H3
0
300
Expression
0
400
0
200
400
600
800
1000
1200
HCP
LCP
ICP
Gene density

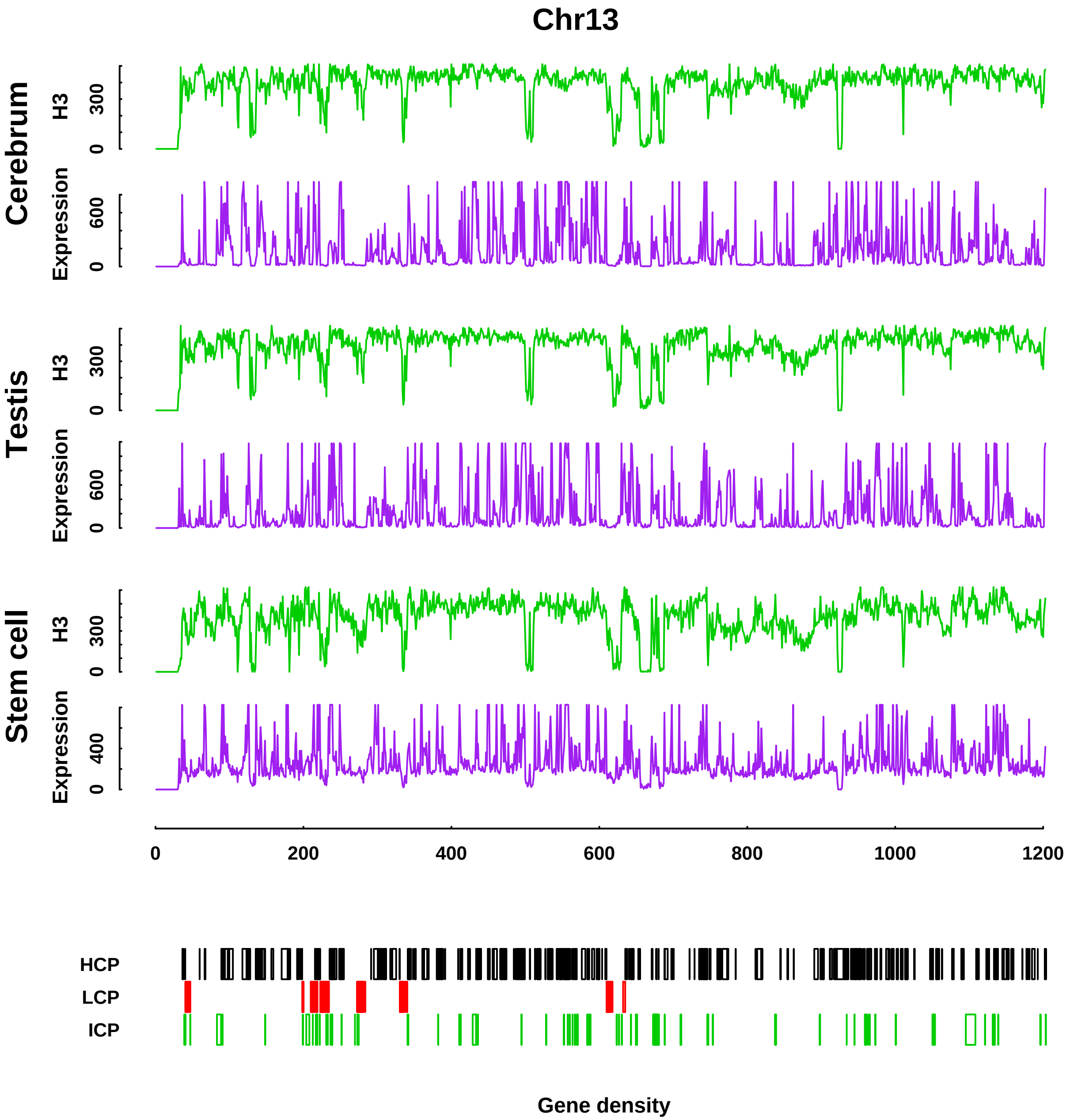

Chr13
Cerebrum
H3
0
300
Expression
0
600
Testis
H3
0
300
Expression
0
600
Stem cell
H3
0
300
Expression
0
400
0
200
400
600
800
1000
1200
HCP
LCP
ICP
Gene density

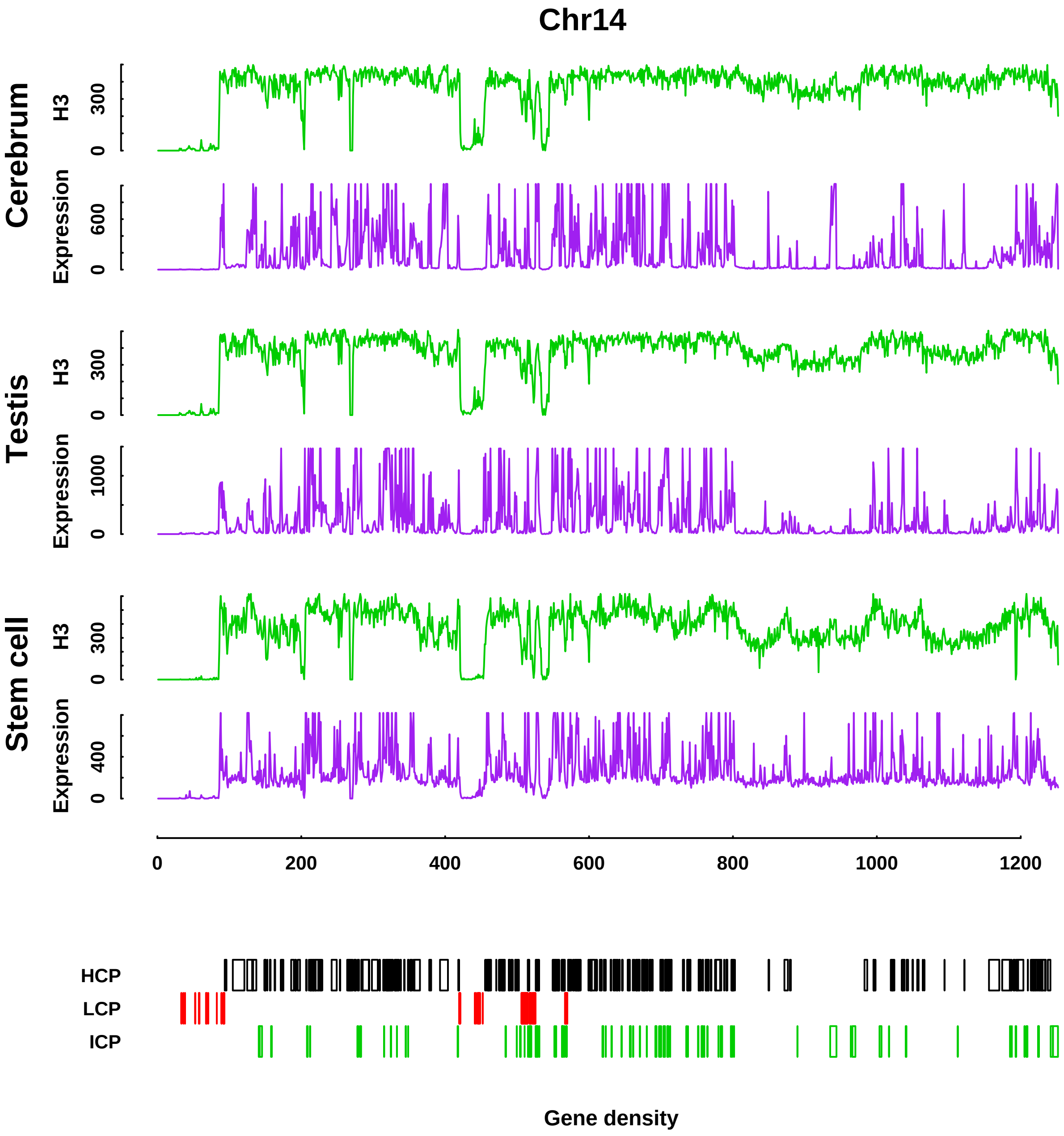

Chr14
Cerebrum
H3
0
300
Expression
0
600
Testis
H3
0
300
Expression
0
1000
Stem cell
H3
0
300
Expression
0
400
0
200
400
600
800
1000
1200
HCP
LCP
ICP
Gene density

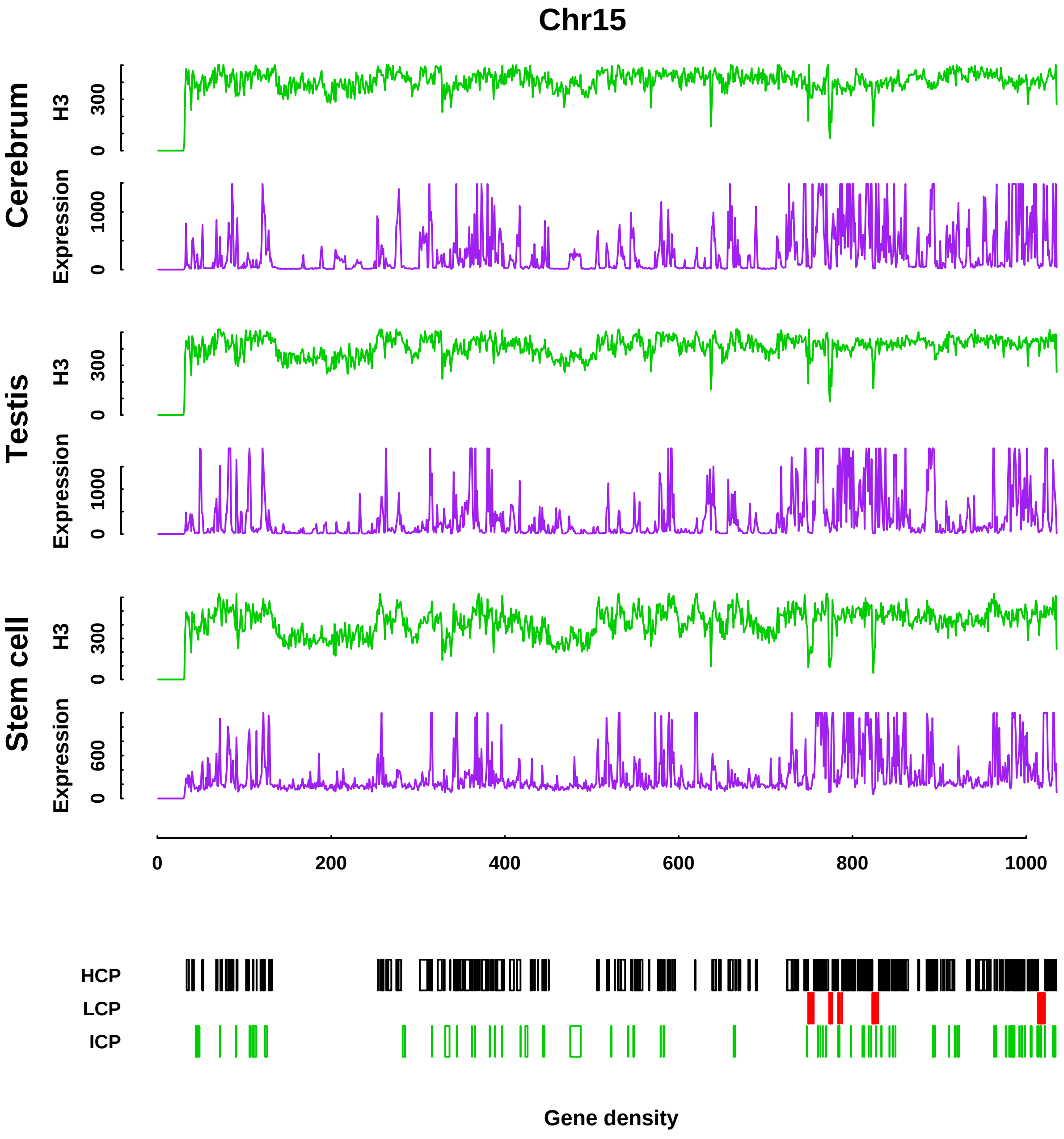

Chr15
Cerebrum
Testis
Stem cell
H3
Expression
0
300
1000
600
0
200
400
600
800
1000
HCP
LCP
ICP
Gene density

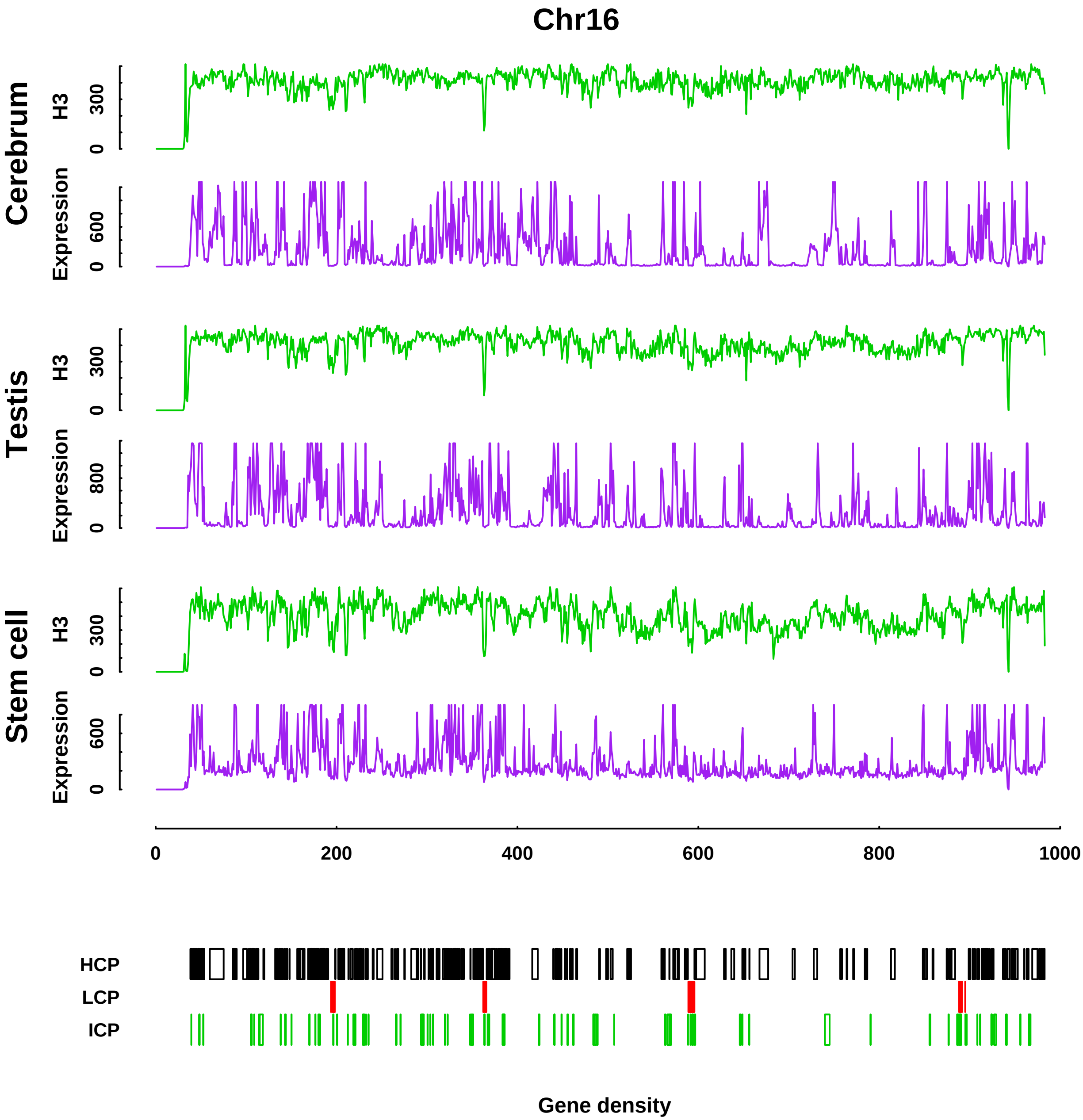

Chr16
Cerebrum
H3
0
300
Expression
0
600
Testis
H3
0
300
Expression
0
800
Stem cell
H3
0
300
Expression
0
600
0
200
400
600
800
1000
HCP
LCP
ICP
Gene density

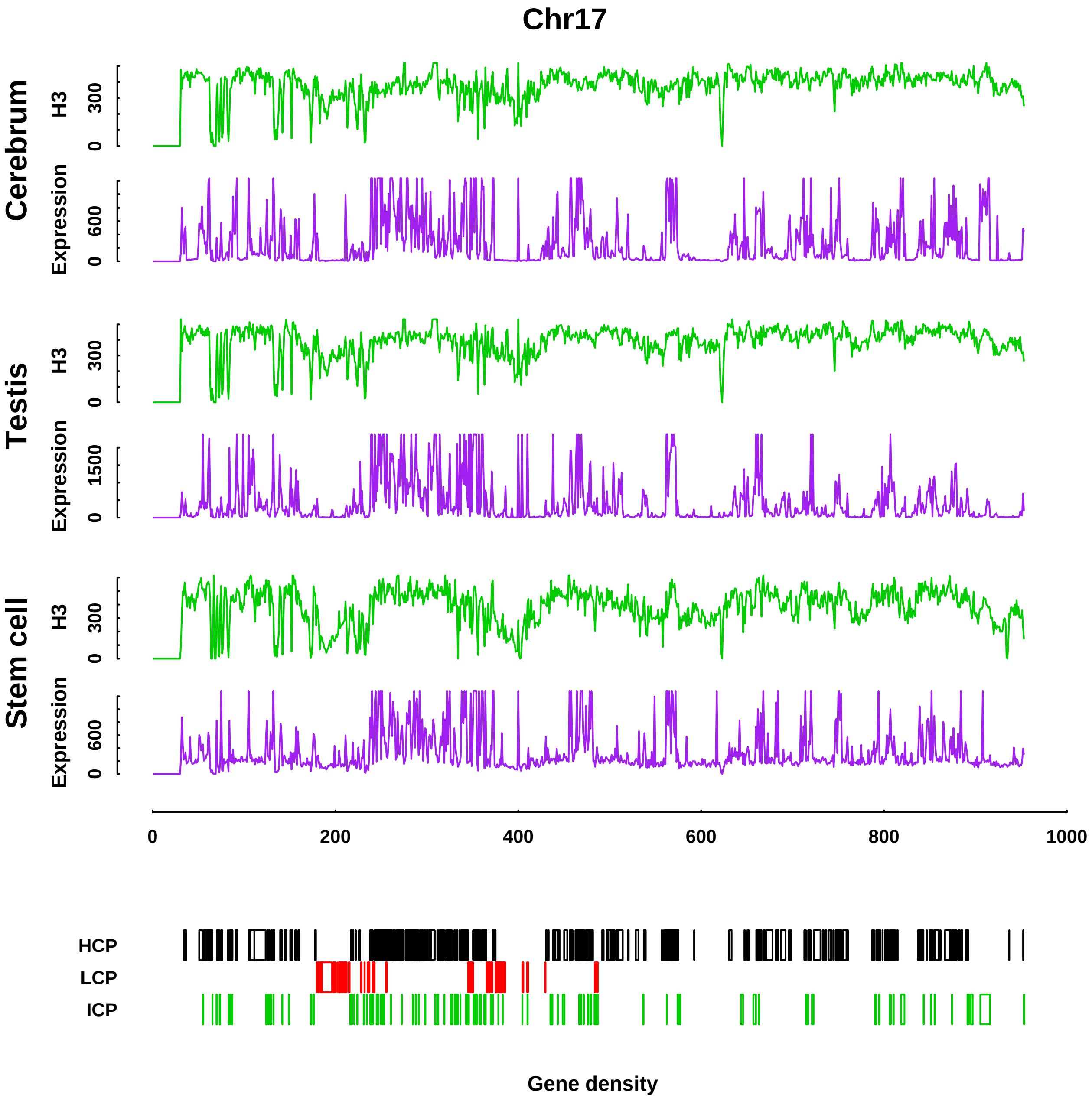

Chr17
Cerebrum
H3
0
300
Expression
0
600
Testis
H3
0
300
Expression
0
1500
Stem cell
H3
0
300
Expression
0
600
0
200
400
600
800
1000
HCP
LCP
ICP
Gene density

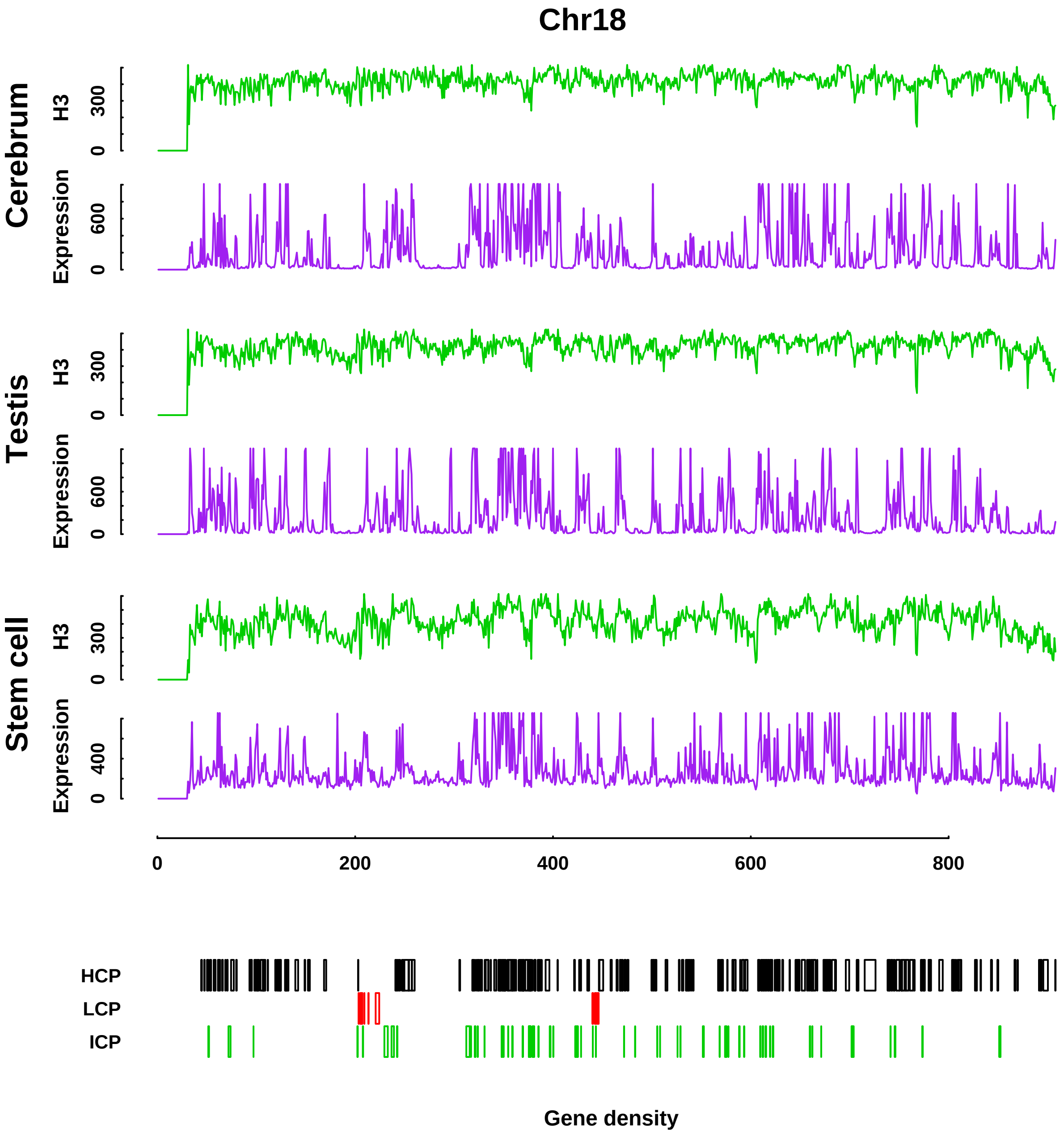

Chr18
Cerebrum
H3
0
300
Expression
0
600
Testis
H3
0
300
Expression
0
600
Stem cell
H3
0
300
Expression
0
400
0
200
400
600
800
HCP
LCP
ICP
Gene density

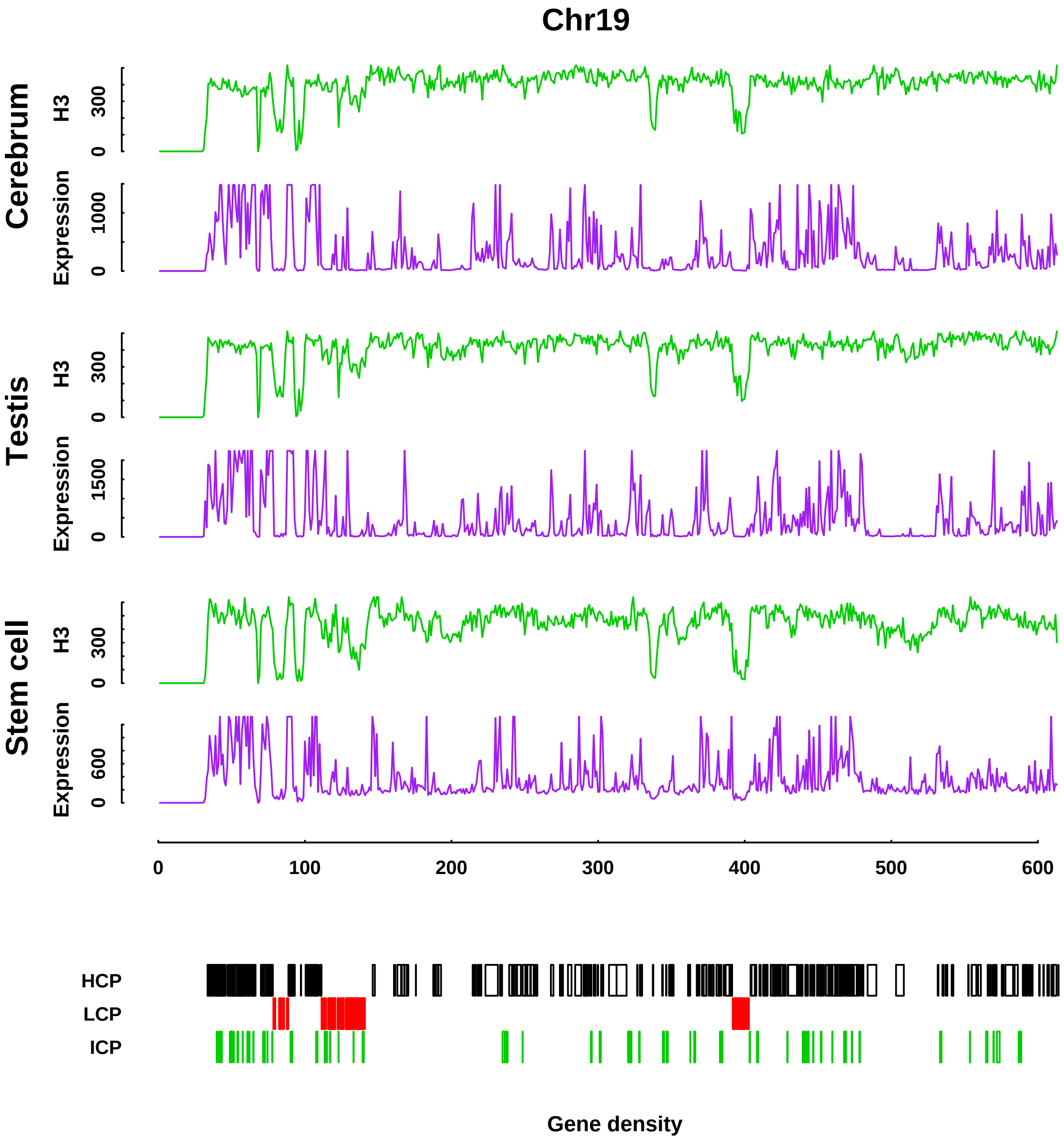
Chr19
Cerebrum
H3
0
300
Expression
0
1000
Testis
H3
0
300
Expression
0
1500
Stem cell
H3
0
300
Expression
0
600
0
100
200
300
400
500
600
HCP
LCP
ICP
Gene density

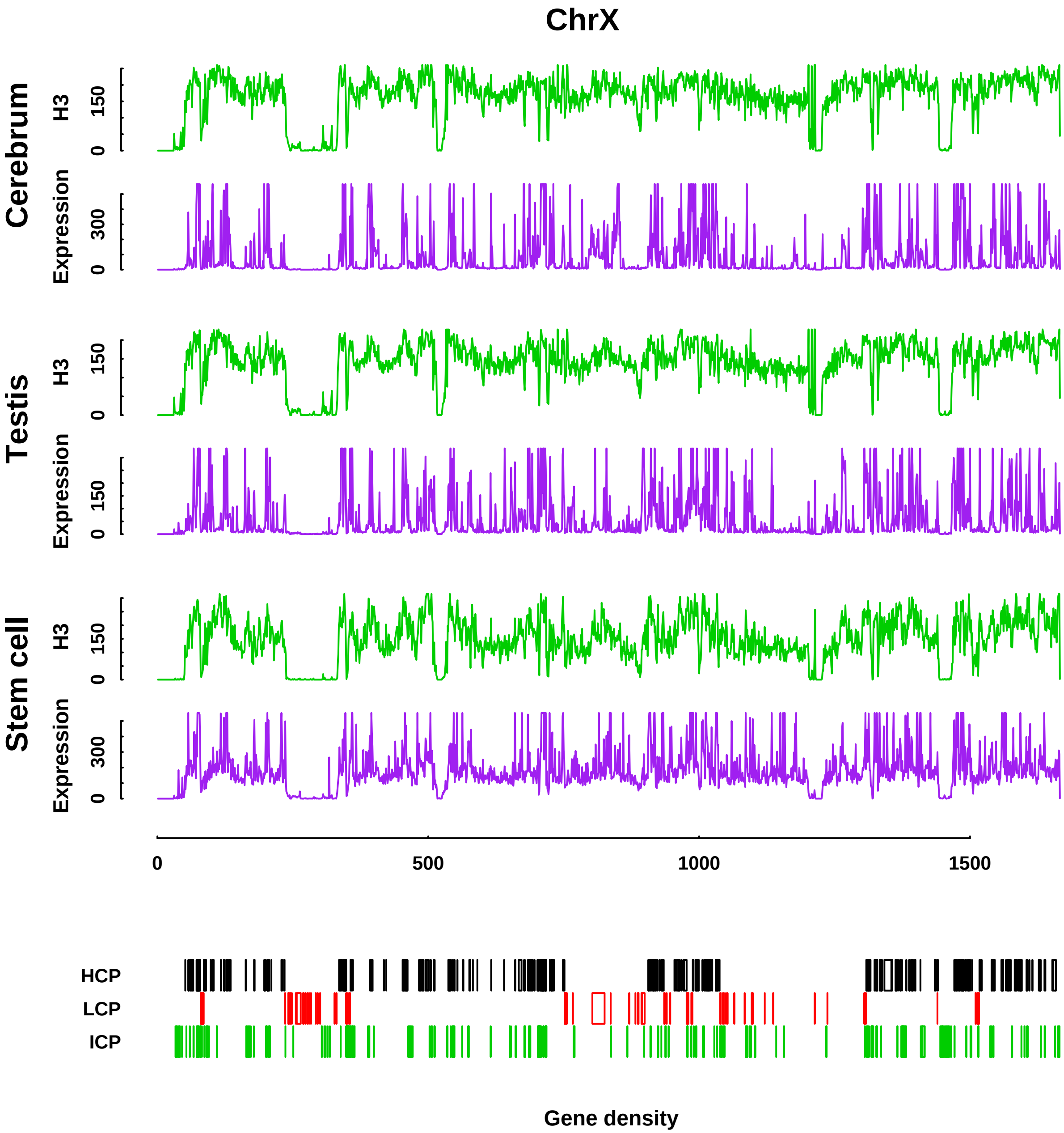
ChrX
Cerebrum
H3
150
0
Expression
300
0
Testis
H3
150
0
Expression
150
0
Stem cell
H3
150
0
Expression
300
0
0
500
1000
1500
HCP
LCP
ICP
Gene density
