# Supplementary material for: The Disequilibrium of Nucleosomes Distribution along Chromosomes Plays a Functional and Evolutionarily Role in Regulating Gene Expression
Source: PLoS One. 2011 Aug 19;6(8):e23219. doi: 10.1371/journal.pone.0023219 (PMC3158759; doi:10.1371/journal.pone.0023219)
Supplement: Table S1 — The number of unique reads from libraries. (DOC) [file pone.0023219.s005.doc]

**Table S1.** Unique read numbers for libraries.

| Library | Type | Unique read Number |
| --- | --- | --- |
| mouse cerebrum | rmRNA-seq | 71,112,582 |
| mouse testis | rmRNA-seq | 143,270,524 |
| mouse stem cell | rmRNA-seq | 21,556,666 |
| mouse cerebrum | H3 ChIP-seq | 25,080,074 |
| mouse testis | H3 ChIP-seq | 27,924,398 |
| mouse stem cell | H3 ChIP-seq | 4,386,914 |
| Human CD4+ T cell | MNase –seq | 154,582,677 |
