# Supplementary material for: The Disequilibrium of Nucleosomes Distribution along Chromosomes Plays a Functional and Evolutionarily Role in Regulating Gene Expression
Source: PLoS One. 2011 Aug 19;6(8):e23219. doi: 10.1371/journal.pone.0023219 (PMC3158759; doi:10.1371/journal.pone.0023219)
Supplement: Table S3 — A list of tissues or cells. (DOC) [file pone.0023219.s007.doc]

**Table S3.** The list of tissues or cells for HK genes research

|  | Tissue/cell type |
| --- | --- |
| 1 | BAT24 |
| 2 | EB |
| 3 | Islet |
| 4 | S129Bcell |
| 5 | S129ES |
| 6 | blastomeres_4cell |
| 7 | brainC57_mRNA |
| 8 | brain_10w |
| 9 | brain_4w |
| 10 | brain_6d |
| 11 | brain_mRNA_10w |
| 12 | liverC57_mRNA |
| 13 | mammary_degr |
| 14 | mammary_lact |
| 15 | mammary_preg |
| 16 | muscle_mRNA |
| 17 | oocyte |
| 18 | ovary_8w |
| 19 | testis_10w |
